# Peer Bullying Victimization Trajectories for Sexually and Gender Diverse Youth from Early Childhood to Late Adolescence

**DOI:** 10.1007/s10964-024-02020-8

**Published:** 2024-06-07

**Authors:** Jingyi Huang, Tessa M. L. Kaufman, Laura Baams, Susan Branje

**Affiliations:** 1 Department of Youth and Family, Faculty of Social and Behavioural Sciences, Utrecht University, Utrecht, The Netherlands; 2 Department of Pedagogy and Educational Sciences, University of Groningen, Groningen, The Netherlands

**Keywords:** Sexually and gender diverse, Peer bullying victimization, Developmental trajectories, Millennium Cohort Study

## Abstract

Sexually and gender diverse (SGD) youth experience more peer bullying victimization than heterosexual, cisgender youth during adolescence, yet the emergence and persistence of these disparities remain underexplored. Also, it is unclear which factors are associated with these disparities across development, and how these disparities are linked to late adolescent health discrepancies. This study utilized the sample from the Millennium Cohort Study in Britain (*N* = 10,080; 51.3% assigned female at birth; *M*_age_ = 2.28, *SD*_age_ = 0.46 at Wave 2), in which 23.74% of youth reported non-heterosexual attraction, 21.59% reported non-heterosexual identity, and 1.08% reported gender identity not in line with the sex assigned at birth. Using latent class growth modeling, four peer bullying victimization trajectories were identified, with early peak (7.2%), late childhood peak (6.3%), adolescence onset (12.8%), and low (73.6%) rates of victimization. SGD youth, compared to heterosexual and cisgender youth, were found to have increased odds of being in the victimization-involved classes, especially the adolescence onset class. The study further revealed that SGD youth reported more mental health and relational difficulties in childhood, which were linked to their heightened risk of longer-lasting victimization. Further, long-term victimization was found to partially account for the disparities in health and well-being for SGD youth in late adolescence. In conclusion, SGD youth were more likely to experience longer-lasting bullying victimization during childhood and adolescence, its related mental and relational vulnerabilities were already established in childhood, and such victimization disparities were further linked to their detrimental health and well-being in late adolescence. The design, hypotheses, and target analyses of the current study were preregistered on 21st April 2023 at https://osf.io/f2zxy.

## Introduction

The lower levels of health and well-being and the higher rates of victimization experienced by sexually and gender diverse (SGD) youth are well documented. Sexually diverse youth include individuals with non-heterosexual orientations, and gender-diverse youth are those whose gender identity differs from the sex assigned to them at birth. Compared to heterosexual, cisgender youth, SGD youth report higher rates of various types of victimization, such as sexual victimization, school violence, bias-based bullying, and general bullying (Myers et al., 2020, for a meta-analysis). Bullying victimization is defined as repetitive and intentional aggressive behaviors characterized by a power imbalance (Olweus, 1999). While bullying victimization has seen a decline over recent decades overall, the disparities based on sexual orientation (Goodenow et al., 2016) and gender identity (Kiekens et al., 2024) have remained. To illustrate, results from US nationally representative data suggested that SGD youth were about twice as likely as heterosexual, cisgender youth to report bullying victimization (Basile et al., 2020; Johns et al., 2020). Such bullying victimization disparities experienced by SGD youth are further linked to their health difficulties, which include mental, behavioral, and sexual health issues (Russell & Fish, 2019). According to the minority stress framework (Meyer, 1995; 2003), SGD youth might also suffer more from *persistent* victimization across prolonged periods of time instead of episodic victimization, which can yield more damage to youth’s health development (Mustanski et al., 2016). Empirical studies indeed showed a higher likelihood of persistent bullying victimization for SGD adolescents (Kaufman et al., 2020). However, it is likely that such disparities already emerge before adolescence (Martin‐Storey & Fish, 2019). Therefore, research needs to move beyond adolescence to compare victimization trajectories between SGD versus heterosexual, cisgender youth starting in early childhood. Moreover, for both scientific and intervention purposes, it is vital to understand which factors are associated with the trajectories of victimization among SGD youth. While the stigma perspective can not fully answer the bullying disparities experienced by SGD youth, combining it with theories and empirical evidence in the general bullying field can help build a more comprehensive understanding of intra- and interpersonal factors that contribute to the development and persistence of victimization. Last, knowledge is needed about the extent to which trajectories of bullying victimization disparities during childhood and adolescence can be related to SGD youth’s detrimental health and well-being in late adolescence. Therefore, the current study aimed (1) to depict persistent bullying victimization from early childhood (age 3) to late adolescence (age 17) using trajectories and identify the disparities between SGD and heterosexual, cisgender youth, (2) to test which mental health and relational factors at different developmental stages are related to SGD youth’s bullying victimization trajectories, and (3) to examine whether bullying victimization trajectories can explain differences in late adolescent (age 17) health and well-being between SGD and heterosexual, cisgender youth.

### Disparities in Persistent Bullying Victimization and Associated Factors from Childhood to Adolescence

In accordance with the minority stress framework (Meyer, 1995; 2003), SGD youth are more likely than heterosexual, cisgender youth to experience peer bullying victimization, because their expressions, behaviors, or identities are considered different from social norms. The bullying literature aligns with this perspective, suggesting that perpetrators strategically target victims who deviate from group norms (Kaufman et al., 2020), and who are therefore less likely to be defended (Ioverno et al., 2022). Such strategies enable perpetrators to easily enhance their status through bullying without running the risk of becoming less popular when faced by defending peers.

Understanding the onset and development of bullying victimization disparities between SGD youth and heterosexual, cisgender youth is crucial. While most of the previous studies addressing victimization disparities for SGD youth focused on adolescence or young adulthood, it is likely that disparities already start during childhood. From a developmental perspective, children could express cross-gender preferences and identities from age 3 onwards (Boskey, 2014), and be aware of their same-sex attraction from age 7 onwards (Mustanski et al., 2014). While the timing of identity exploration among SGD individuals was found to vary (Lamb & Plocha, 2014), youth who later identified as SGD would already recognize themselves different from the majority social norm in childhood (Mustanski et al., 2014; Savin-Williams & Cohen, 2007). Such early recognition might make them feel under- or misrepresented and marginalized (Suen et al., 2020), and they may already perceive negative attitudes from others (Horn, 2006). These early, often ambiguous experiences may contribute to their heightened vulnerability to bullying victimization during childhood. In support of this, disparities in peer bullying victimization were already reported around late childhood (Mittleman, 2019). While it is important to tackle the prospective development of victimization disparities, youth might not be able or feel comfortable to report an SGD identity as early as disparities emerge. Thus, a common strategy to study the development of SGD youth is the combination of prospective and retrospective methods: Follow participants prospectively, identify SGD status later in adolescence, and then link the adolescents’ SGD status back to their earlier life experiences. For instance, this approach was used to address the developmental disparities of victimization (Kaufman et al., 2020; Martin‐Storey & Fish, 2019) and mental health (La Roi et al., 2016; Pachankis et al., 2022).

Although it has been proposed that minority stress accounts for SGD disparities in persistent victimization (Meyer, 1995; 2003), more specific factors associated with such persistence are still unclear. This study proposes two types of factors that might be related to the persistence of bullying victimization among SGD youth: mental health and relational factors. Beyond present stigma that can explain victimization disparities, socioemotional vulnerabilities resulting from SGD youth’s previous stigmatized experiences can also heighten their risk of bullying victimization in the future. Further, with the co-occurrence of victimization, interpersonal rejection, and psychological distress, SGD youth are likely to be trapped in a vicious circle that underlies the persistence of victimization (Zimmer-Gembeck, 2016). Regarding *mental health*, youth with low self-esteem and internalizing problems are less likely to defend themselves, which emboldens bullies and makes them believe they will not be punished (Reijntjes et al., 2010; Storch & Ledley, 2005). Also, youth with mental health symptoms are less likely to escape from victimization due to the tendency to develop maladaptive coping styles (Borecka-Biernat, 2020). Thus, while stigmatized experiences can lead to a range of mental health issues among SGD youth, including more internalizing problems (Espelage et al., 2018; Mustanski et al., 2016) and lower self-esteem (Russell & Fish, 2016), these symptoms might exacerbate the vulnerability of SGD youth to bullying. In addition, SGD youth are more likely to show hyperactive/inattention symptoms (e.g., Goetz & Adams, 2022), which have been related to victimization (Wiener & Mak, 2009). Youth with hyperactive/inattention symptoms are likely to develop emotional and peer problems (Hoza et al., 2005; Rosen & Factor, 2015), which further increase their risk of peer victimization (Fogleman et al., 2016). Given the generally stable nature of these symptoms, it is likely that these symptoms are associated with higher persistent victimization risks. In sum, emotional problems, low self-esteem, and hyperactive/inattention problems might act as potential mechanisms for SGD youth’s longer-lasting victimization.

Besides these mental health factors, *relational* factors may also serve as possible mechanisms that are associated with persistent victimization disparities. The generalized unsafety framework (Diamond & Alley, 2022) emphasizes the absence of social safety, such as a lack of interpersonal closeness and support, as a serious obstacle for SGD youth. Among different interpersonal contexts, family and peer relations are two vital aspects of youth’s development. In the family context, parent-child relationships among SGD youth are characterized by more negative features, such as hostility, rejection, and less support (Diamond & Alley, 2022; Montano, et al., 2018). One reason for this observation might be that parents show greater discomfort when their children do not conform to general gender norms (Spivey et al., 2018), which translates to a less supportive parent-child relationship for SGD children. Negative parent-child relationship quality could be related to a higher risk of persistent victimization through the increase in children’s rejection sensitivity (Gao et al., 2021). Regarding the peer context, SGD youth often experience more negative or fewer positive peer connections than heterosexual, cisgender youth (Diamond & Lucas, 2004), likely because they are considered different from or inferior to dominant social norms around gender and sexuality (Harkness & Israel, 2018). Since bullies typically target isolated or rejected peers, SGD youth seem at higher risk of experiencing victimization across ages and contexts. Thus, SGD youth might be at risk of getting stuck into vicious or few supportive interpersonal relations across different contexts. Altogether, it is likely that more negative parent-child and peer relationships, including a lack of social support, might be associated with SGD youth’s higher risk of persistent bullying victimization compared to heterosexual, cisgender youth.

### Late Adolescent Health and Well-Being Related to SGD Youth’s Persistent Bullying Victimization Experiences

In addition to understanding the mental and relational factors that are associated with the persistence of victimization, it is also important to understand how trajectories of peer bullying victimization are related to the health and well-being discrepancies for SGD youth during late adolescence. Late adolescence serves as a pivotal developmental period when youth prepare for important role transitions to adulthood. The well-being of SGD youth in late adolescence is particularly important as marginalized identities can introduce obstacles during this transitional phase (Zarrett & Eccles, 2006), and health and well-being challenges can also delay youth’s maturity and development (Skehan & Davis, 2017). Beyond bullying victimization disparities at specific time points, it is likely that the developmental disparities across childhood and adolescence result in health differences between SGD youth and heterosexual, cisgender youth in late adolescence. A dose-response association could exist in the effects of victimization so that the damage of victimization cumulates over time (Manly et al., 1994; Musicaro et al., 2019), and thus, long-term exposure to victimization can cause more harm to youth’s development than episodic experiences. While mean-level victimization disparities cannot fully account for SGD youth’s lower health and well-being (Martin‐Storey & Fish, 2019), disparities in heterogeneous victimization trajectories may provide a better explanation for these observed discrepancies.

### A Comprehensive Measurement of SGD Disparities: Differentiating Between Subgroups and Informants

When studying disparities for SGD youth, a more individualized perspective is necessary beyond the general assessments of mean-level disparities between SGD versus heterosexual, cisgender populations, because SGD is a broad, multidimensional construct, and sexual orientation (SO) and gender identity (GI) each also include different subgroups (Baams & Kaufman, 2023; Espino et al., 2024).

First, it is commonly recognized that SO and GI are two independent constructs. The former one is associated with sexual and/or romantic interpersonal experiences, while the latter one concerns how individuals experience/express and identify their gender. A review of population-based studies showed that around 10.6% to 17.2% of adolescents were sexually diverse, and around 1.6 to 3% of adolescents were gender diverse (Baams & Kaufman, 2023). Moreover, SO is a multidimensional construct consisting of several independent aspects, including attraction, identification, and behavior (Mustanski et al., 2014). Conceptually, these aspects differ; for example, attraction refers to one’s emotional, romantic, and sexual feelings towards other people, behavior relates to the gender of one’s romantic and/or sexual partners, and sexual identity is an individual’s self-identification that relates to attractions, behaviors, and relationships in a given cultural context. Empirical evidence supported the multidimensional nature of SO, showing only weak correlations among these SO dimensions (Priebe & Svedin, 2013). Additionally, a study reported a mere 15% overlap among all three dimensions, while about one-third overlap existed between sexual attraction and sexual identity (Igartua et al., 2009). Therefore, it is recommended to identify sexually diverse youth based on different dimensions, independently (Baams & Kaufman, 2023; Mustanski et al., 2014). In adolescence, the “behavior” dimension may be less effective due to limited experiences with sexual behavior and intimate relationships at this age (Priebe & Svedin, 2013), and sexual attraction (e.g., Martin-Storey & Fish, 2019) and sexual identity (e.g., Poteat et al., 2021) are more common identifiers while most studies used solely one of them.

Another important, yet often overlooked, differentiation is the gradients in sexual orientation. Youth reporting to be mainly heterosexual or mostly other-sex attracted are often considered part of the broader bisexual population, yet some studies included them as part of the heterosexual group (e.g., Xu & Rahman, 2022). However, the mainly heterosexual or mostly other-sex attracted group has been shown to represent a distinct sexual orientation subgroup, exhibiting unique sexual-related behavior patterns that differentiate them from exclusively heterosexual and other sexually diverse groups such as bisexual and lesbian (Thompson & Morgan, 2008). While retrospective studies indicated that mainly heterosexual individuals reported more childhood victimization than exclusively heterosexual individuals (Talley et al., 2016; Zou & Andersen, 2015), doubts were raised regarding how and to what extent this group experiences minority stress, given their less visible deviation from heteronormativity (Vrangalova & Savin-Williams, 2014). In sum, as findings are mixed, the sexually diverse group and the mainly heterosexual/mostly other-sex attracted group cannot be considered identical.

In addition, when studying early disparities for SGD youth, it is unclear which informant(s) can provide the most valid information about socio-emotional experiences. Parents typically report on social and emotional experiences in early childhood when children’s reflective abilities are limited (Darling-Churchill & Lippman, 2016). However, SGD youth may not share as much information with their parents as heterosexual, cisgender youth do, as they frequently experience unsafe family environments (Diamond & Alley, 2022). Fearing rejection from parents, adolescents often conceal their SGD experiences (Pasek et al., 2017) which may prevent parental awareness of socioemotional difficulties related to stigma. Consequently, relying solely on parent-reported information may underestimate disparities. For instance, although parents could detect disparities in internalizing problems between sexually diverse and heterosexual adolescents, it could be less sensitive compared to youth-reported data (Kaufman et al., 2020). While limited studies have addressed parents’ perceptions of SGD youth’s emotional and relational experiences in addition to youth reports, understanding these perceptions is crucial, because parents, as one of the most important social resources of SGD youth, may fail to provide timely support if they do not accurately perceive youth’s experiences (Baams, 2019).

## Current Study

While bullying victimization disparities between SGD and heterosexual, cisgender youth have been well-documented, further knowledge is needed regarding its persistence and early development. Besides, knowledge is lacking on how SGD youth’s mental and relational vulnerabilities relate to victimization trajectories, and how such heterogeneous developmental disparities are linked to well-being in late adolescence. The current study followed youth from early childhood to late adolescence, aiming to capture disparities in persistent bullying victimization by identifying heterogeneous trajectories using latent class growth models (LCGM) and exploring disparities in trajectories between SGD and heterosexual, cisgender youth (Aim 1), test mental health and relational factors that might be related to SGD youth’s victimization trajectories (Aim 2), and examine whether victimization trajectories can explain differences in SGD youth’s health and well-being in late adolescence (Aim 3). It was expected that SGD youth would show higher risks of being classified in victimized trajectories with long-term exposure compared to heterosexual, cisgender youth, and that such disparities might emerge in childhood (Hypothesis 1), mental health issues including emotional problems, self-esteem, and hyperactive/inattention symptoms, and relational factors including parent-child closeness, peer problems, and social support from peer and family would be associated with SGD youth’s longer-lasting victimization experiences (Hypothesis 2), and victimization trajectory disparities would explain health and well-being discrepancies between SGD and heterosexual, cisgender youth in late adolescence including emotional problems, health, self-harm, and substance use (Hypothesis 3). This study treated SGD as a multidimensional construct, by including both sexual attraction and sexual identity to identify sexually-diverse youth comprehensively, and treated these two dimensions separately to explore potential discrepancies between sexual-attraction diverse and sexual-identity diverse youth. In line, the “completely” and “mainly” sexually diverse groups were treated as different populations. Last, to acquire a more comprehensive understanding of disparities, this study relied on both parent- and self-reported data on mental and relational factors. The design, hypotheses, and target analyses of the current study were pre-registered (https://osf.io/f2zxy).

## Methods

### Participants

The current sample came from the ongoing Millennium Cohort Study (MCS) project, a multi-informant longitudinal birth cohort study following a nationally representative sample of 19,244 families with children born in the United Kingdom (UK) around the turn of the century. Families that participated in the MCS were randomly selected with a stratified design to cover the disadvantaged and ethnically diverse areas to reach representativeness. So far, the MCS has released seven measurement waves of data starting from year 2001, capturing information from when participants were 9 months (wave 1, W1), 3 years (wave 2, W2), 5 years (wave 3, W3), 7 years (wave 4, W4), 11 years (wave 5, W5), 14 years (wave 6, W6), and 17 years old (wave 7, W7) (Connelly & Platt, 2014).

Data used in the current study were reported by youth and the main caregivers (mostly mothers) via home visits, postal, and web surveys. Participants’ consent was required at each wave. Youth’s SO and GI were reported by youth at age 17 (W7), where the valid sample size was *n* = 10,345. For the purposes of this study, participants were included if they had (1) provided at least one valid response on the SO/GI items at W7, and (2) the main caregiver reported at least one valid response on the victimization items across W2 to W7. Data from participants who did not meet the above conditions were excluded (*n* = 265). Finally, *N* = 10,080 youth (51.3% assigned female at birth) were selected as the current analytic sample, among whom 78.4% were White, 7.9% were Pakistani or Bangladeshi, 4% reported being mixed race, 4% were Black or Black British, 2.9% were Indian, and 2.8% were Chinese or reported another ethnicity. Most of the participants were born in 2001 (70.6%), while 28.9% were born in 2000, and 0.5% in 2002. Attrition analysis was performed by conducting t-tests for standardized family income and parental education level at W2, and by conducting a Chi-square test for youth’s ethnicity. Results showed that the dropped-out families had significantly lower standardized family income (*t* = 12.47, *df* = 13274, *p* < 0.001), lower level of parental education (*t* = 20.27, *df* = 14763.46, *p* < 0.001), and were more likely to be White than families of color (*χ*² = 43.03, *df* = 1, *p* < 0.001). In total, 10,080 cases were included in the analyses, in which missing rates on victimization items from W2 to W7 were 16.7%, 12.2%, 13.1%, 10.2%, 11.2%, and 22.0%, respectively.

### Measurement

#### Sexual orientation

Two dimensions of sexual orientation were reported by youth at W7, namely sexual attraction and sexual identification. Sexual attraction was assessed by one item “I have felt sexually attracted…” Three groups were identified: heterosexual (75.76%, *n* = 7637), mostly other-sex attracted (13.67%, *n* = 1378), and sexual-attraction diverse (10.07%, *n* = 1015). Heterosexual youth were those who answered *attracted only to the opposite sex*, mostly other-sex attracted youth were those who answered *mostly attracted to the opposite sex but at least once to the same sex*, and sexual-attraction diverse youth included youth who answered *attracted about equally often to opposite sex and the same sex* (*n* = 384), *mostly to the same sex* (*n* = 194), *only to the same sex* (*n* = 135), *never felt sexually attracted to anyone* (*n* = 291), and *don’t know* (*n* = 11). Fifty participants in the final sample did not provide valid responses to this item. The different sexual-attraction diverse subgroups were collapsed into the broader category “sexual-attraction diverse” because the sample sizes of the subgroups were too small to reliably analyze separately.

Sexual identity was assessed by one item: “Which of the following options best describes how you currently think of yourself?” and three groups were identified: heterosexual (78.06%, *n* = 7868), mainly heterosexual (10.89%, *n* = 1098), and sexual-identity diverse (10.70%, *n* = 1076). Heterosexual youth were those who answered *exclusively heterosexual/straight*, mainly heterosexual youth were those who answered *mainly heterosexual*, and sexual-identity diverse youth included youth who answered *bisexual* (*n* = 655), *mainly gay or lesbian* (*n* = 90), *completely gay or lesbian* (*n* = 158), *other* (*n* = 157), and *don’t know* (*n* = 16). Thirty-eight participants in the final sample did not provide valid responses to this item. Same as mentioned in sexual attraction, the different sexual-identity diverse subgroups were collapsed together.

#### Sex assigned at birth

Youth’s sex assigned at birth was reported by parents with 1 = *male* and 2 = *female*.

#### Gender identity

Youth’s self-reported gender identity was assessed at W7 by one item: “Which of the following options best describes how you currently think of yourself?” After cross-checking youth’s gender identity with their sex assigned at birth, most of the participants were coded as cisgender (98.34%, *n* = 9913), and 1.08% were collapsed as *gender diverse*, including *transgender* (*n* = 48), *non-binary* (*n* = 21), *androgynous* (*n* = 8), *gender fluid* (*n* = 9), and *other gender* (*n* = 20). Sixty-one participants in the final sample did not provide valid responses to this item.

#### Emotional problems (Youth-reported, W4-W7)

Youth-reported emotional problems (W4-W6) were assessed using different, age-appropriate measures at different ages. Emotional problems at W4 were measured by seven moods (e.g., “How often do you feel sad”) on a 3-point scale from 1 = *never* to 3 = *all of the time*, and emotional problems at W5 were measured by six moods (e.g., “How often do you feel sad”) on a 5-point scale from 1 = *never* to 3 = *almost always*. Confirmatory factor analyses (CFAs) were tested (see Table S1) and two items at W4 (quiet and laugh) and one item at W5 (laugh) were dropped due to low factor loadings (lower than 0.3). The retained five items showed satisfied factor scores for both W4 and W5, while Cronbach’s *α* was 0.50 for W4 and 0.72 for W5. Thus, the factor score for W4 and the mean score for W5 were used for further analysis. Emotional problems were measured by the 13-item Short Mood and Feelings Questionnaire (SMFQ, Angold et al., 1995) (e.g., “In the past two weeks I felt miserable or unhappy”, from 1 = *not true* to 3 = *true*) at W6, and by the 6-item Kessler 6 (K6; Kessler et al., 2002) scale (e.g., “During the last 30 days, about how often did you feel hopeless”, 1 = *none of the time* to 5 = *all of the time*) at W7. Cronbach’s *α*s in the current study were 0.93 for SMFQ and 0.86 for K6, and their mean scores were calculated for further analysis.

#### Self-esteem (Youth-reported, W5-W6)

Youth-reported self-esteem was assessed at W5 and W6 by the 5-item short version of the Rosenberg self-esteem scale (e.g., “I feel good about myself”) (Rosenberg, 1989). Youth reported on a 4-point scale from 1 = *strongly disagree* to 4 = *strongly agree*. Cronbach’s *α*s ranged from 0.74 to 0.91 in the current study. Mean scores were calculated for further analysis.

#### Peer relationships (Youth-reported, W4-W6)

Youth’s self-reported peer relationships included the availability of friendships and peer exclusion. Availability of friendships (W4-W6) was measured by one item at W4: “How many friends do you have” (3-point, from 1 = *not many* to 3 = *lots*), one item at W5 “When you are not at school, how often do you spend time with your friends” (6-point, from 1 = *most days* to 5 = *never*, and 6 = *don’t have any friends*; reversed), and two items at W6 “Do you have any close friends” (1 = *yes*, 2 = *no*) and “When you are not at school, how often do you spend time with your close friends” (5-point, from 1 = *never* to 5 = *most days*). The two items at W6 were merged by recoding youth responding *do not have close friends* as 0. Youth’s self-reported peer exclusion was measured by one item at W4: “How often do you feel left out of things by other children” (3-point, from 1 = *never* to 3 = *all of the time*).

#### Parent-child closeness (Youth-reported, W6)

Youth self-reported on parent-child closeness was measured at W6 using two items “Overall, how close would you say you are to your mother/father” rated on a 3-point scale from 1 = *fairly close* to 3 = *extremely close*. The mean score of the two items was calculated to represent youth’s general closeness with their father and mother.

#### Social support (Youth-reported, W4-W6)

Youth-reported social support was conceptualized in two ways: disclosure and available support. Disclosure of problems was reported by youth using one item with multiple response options: “What do you do if you are worried about something”. At W4, youth responded on: *keep it to myself*, *tell a friend*, *tell someone at home*, and *tell a teacher*. At W5, youth responded on: *keep it to myself*, *tell a friend*, *tell someone at home*, *tell a teacher*, and *tell someone else*. At W6, youth responded on: *keep it to myself*, *tell my parent(s)*, *tell a brother or sister*, *tell a friend or boyfriend/girlfriend*, *tell another relative*, *tell a teacher*, and *tell another adult*. Dummy variables were created at each wave, with youth reporting at least one social resource as 1, otherwise as 0.

Available social support from family and peers was measured by three items at W6: (1) *I have family and friends who help me feel safe, secure and happy*; (2) *There is someone I trust whom I would turn to for advice if I were having problems*; and (3) *There is no one I feel close to* (reverse). Youth reported their perceived social support on a 3-point scale from 1 = *not true at all* to 3 = *very true*. Cronbach’s *α* was 0.6 in the current sample. Mean scores were calculated, with a higher score representing more perceived social support.

#### Health (Youth-reported, W7)

Youth-reported health was measured by two items at W7. Youth’s general health was measured by one item “How would you describe your health generally” rating on a 5-point response (1 = *poor* to 5 = *excellent*). Social and behavioral concerns were measured by first asking “Do you have any physical or mental health conditions or illnesses lasting or expected to last 12 months or more? (0 = *no*, 1 = *yes*)”, youth who answered yes would then respond on “social or behavioral conditions or illnesses (for example associated with autism, attention deficit disorder or Asperger’s Syndrome)” based on a binary response (0 = *no*, 1 = *yes*). Youth who answered *yes* to the second question were coded as 1 (have mental health conditions or illnesses), and those who answered *no* for both questions were coded as 0.

#### Self-harm (Youth-reported, W7)

Youth-reported self-harm behaviors (W7) were measured by six items (e.g., “Cut or stabbed yourself”) on a binary response (0 = *no* and 1 = *yes*). The Cronbach’s *α* was 0.7 in the current sample. A sum score was calculated, with a higher score representing more self-harm behaviors.

#### Substance use (Youth-reported, W7)

Youth-report substance use was reported at W7 including (1) smoking: “Which of the following best describes you” (1 = *I have never smoked cigarettes* to 6 = *I usually smoke more than six cigarettes a week*); (2) vaping: “Which of the following best describes you” (1 = *I have never tried an e-cigarette or vaping device* to 6 = *I usually use an e-cigarette or vaping device more than six times a week*); (3) alcohol use: “Have you ever had an alcoholic drink?” (0 = *no* and 1 = *yes*), and “How many times have you had an alcoholic drink in the last 4 weeks” (1 = *never* to 7 = *40 or more times*); and (4) cannabis use: “Have you ever taken cannabis” (0 = *no* and 1 = *yes*) “In the past year how many times have you taken Cannabis” (1 = *not taken in last year* to 5 = *more than 10 times*). The two items of alcohol use were merged into one item by coding youth who never ever had an alcoholic drink as 0. Also, the same coding was conducted for cannabis use. Then, cut-offs were set to identify frequent users with youth who smoked or vaped more than 6 times a week, had alcoholic drinks at least 6 times in the past four weeks, and used cannabis at least 3 times in the past years.

#### Peer bullying victimization (Parent-reported, W2-W7)

Parent-reported youth’s peer bullying victimization (W2-W7) was measured using one item extracted from the Strengths and Difficulties Questionnaire (SDQ; Goodman & Goodman, 2011): “Picked on or bullied by other children” responding on a 3-point scale from 1 = *not true* to 3 = *very true*. The validity of parent-reported bullying victimization was reported in Appendix N testing the consistencies compared with youth’s self-reported victimization.

#### Emotional problems of the child (Parent-reported, W2-W6)

Parent-reported youth’s emotional problems (W2-W6) were measured by the 5-item emotional symptoms subscale (e.g., “Often seems worried”) of the SDQ from W2 to W6. Youth’s parents reported on a 3-point scale from 1 = *not true* to 3 = *very true*. SDQ had been tested to show acceptable validity, reliability, and measurement invariance (Kersten et al., 2016, Murray, et al., 2020), and its psychometric features in the MCS had been proven to be adequate (Croft et al., 2015). The Cronbach’s α’s of the emotional problems subscale of the current sample ranged from 0.51 to 0.72, and were lower than 0.60 at W2 and W3. Since the CFA showed a satisfied factor structure (see Table S2), factor scores of emotional problems were saved and used for further analysis.

#### Hyperactive/inattention symptoms (Parent-reported, W2-W6)

Parent-reported youth’s hyperactive/inattention symptoms were measured by the 5-item hyperactive/inattention subscale (e.g., “Constantly fidgeting”) of SDQ from W2 to W6. Youth’s parents reported on a 3-point scale from 1 = *not true* to 3 = *very true*. Cronbach’s *α*s ranged from 0.71 to 0.79 in the current study. Mean scores were calculated for further analysis.

#### Peer problems (Parent-reported, W2-W6)

Parent-reported peer problems were measured from W2 to W6 by four items from the peer relationship problems subscale from the SDQ (e.g., “Generally liked by other children (reverse)”), excluding the item on peer victimization. Parents reported youth’s peer problems on a 3-point scale from 1 = *not true* to 3 = *very true*. The Cronbach’s *α*s of the rest four items ranged from 0.46 to 0.57 in the current study. The low *α* might be caused by the relatively low number of items. The CFA results showed a satisfied factor structure (see Table S2), thus the factor scores of peer problems were used for further analysis.

#### Parent-child closeness (Parent-reported, W3-W6)

Parent-reported parent-child closeness (W3-W6) was reported by parents using one item “Overall, how close would you say you are to your child” on a 4-point scale from 1 = *not very close* to 4 = *extremely close*.

#### Covariates (Parent-reported)

Demographic variables were considered as covariates in the current study, including net family income, parents’ educational level, youth’s ethnicity, and age. Net family income was reported by parents on W2, and the standardized income of couple families and lone-parent families was used. Parents’ educational levels were measured by “*NVQ equivalent of highest Academic qualification*” at W1 and W2. Youth’s ethnicity was reported by parents, and a dummy variable was created (*0 = white* and *1 = not white*). Youth’s age was calculated based on the parent-reported year of birth.

### Data Analysis

Statistical models were tested using Mplus (version 8.9). To handle models with missing data, Full Information Maximum Likelihood (FIML) analyses in Mplus were employed for all model estimations.

First, to test the heterogeneity in peer bullying victimization development, a series of LCGMs were conducted with Maximum Likelihood estimation with robust standard errors (MLR), and varied numbers of trajectories were specified. The best fitting solution was chosen based on (1) lower Akaike Information Criterion (AIC) score and Sample Size Adjusted Bayesian Information Criterion (aBIC) score; (2) significant adjusted Lo-Mendell-Rubin likelihood test (Adj-LMR-LRT) and bootstrap likelihood ratio tests (BLRT); (3) acceptable entropy (>0.70); and (4) acceptable average latent class probabilities (>0.70) (Wickrama et al., 2016). Also, the smallest class should ideally cover over 5% of the whole sample given the relatively small proportions of SGD youth in the sample. In addition, the theoretical justification and interpretability of the trajectories were considered (Nagin & Odgers, 2010).

Second, disparities between SGD and heterosexual, cisgender youth in bullying victimization trajectories were examined by testing the predictive roles of SGD statuses for class membership. The class membership extracted in the first step was used as a nominal indicator of victimization, and Montecarlo integration was applied to analyze the nominal outcome. Specifically, three models with sexual attraction, sexual identity, and gender identity as predictors of class membership were tested separately. Deviating from the pre-registered analysis, in steps 2 and 3, the class membership was used to test victimization disparities (see Appendix B for details).

Third, the mediating roles of the proposed mental health and relational factors related to SGD youth’s bullying victimization trajectories were tested. The class membership was used as the nominal indicator of victimization. A series of models via different mediators between SGD status and victimization class membership were tested (Appendix C, Fig. S1a). The proposed factors included: emotional problems, self-esteem, hyperactive/inattention problems, peer problems, peer relationships, parent-child closeness, and social support. Because the proposed factors were not measured consistently across waves, to keep the interpretation of the results across waves consistent, earlier waves of associated factors were not controlled. Also, to reduce the risk of false discovery rates (FDR), the *α* levels were adjusted using the Benjamini-Hochberg procedure (Benjamini & Hochberg, 1995) based on the number of waves a particular factor was measured.

Fourth, the effects of SGD status and bullying victimization trajectories on late adolescents’ health and well-being were tested. Several mediation models of SGD status on health and well-being factors via posterior probabilities of victimization trajectories were tested (Appendix C, Fig. S1b). Health and well-being variables included health, emotional problems, self-harm behaviors, and substance use.

Net family income, parents’ educational level, and youth’s ethnicity, age, and sex assigned at birth were included as covariates from the second to fourth steps of analyses. Missingness on covariates was handled by modeling the variances under the MODEL command.

## Results

### Preliminary Analysis

The overlap between sexual attraction and sexual identity, and the crosstabulation among SO/GI subgroups are reported in Appendix D. Tables S6–S9 (Appendix E) report the descriptive statistics and correlations among key study variables. Most of the variables were significantly correlated with each other. Sexually diverse youth (both by sexual attraction and sexual identity) reported higher levels of victimization than heterosexual youth since middle childhood and gender diverse youth reported higher levels of victimization than cisgender youth since early adolescence. Longitudinal measurement invariance was tested when study variables were measured with a consistent scale across time (Table S10). Metric invariance was confirmed for all tested models.

### Victimization Trajectories

**Fig. 1:**
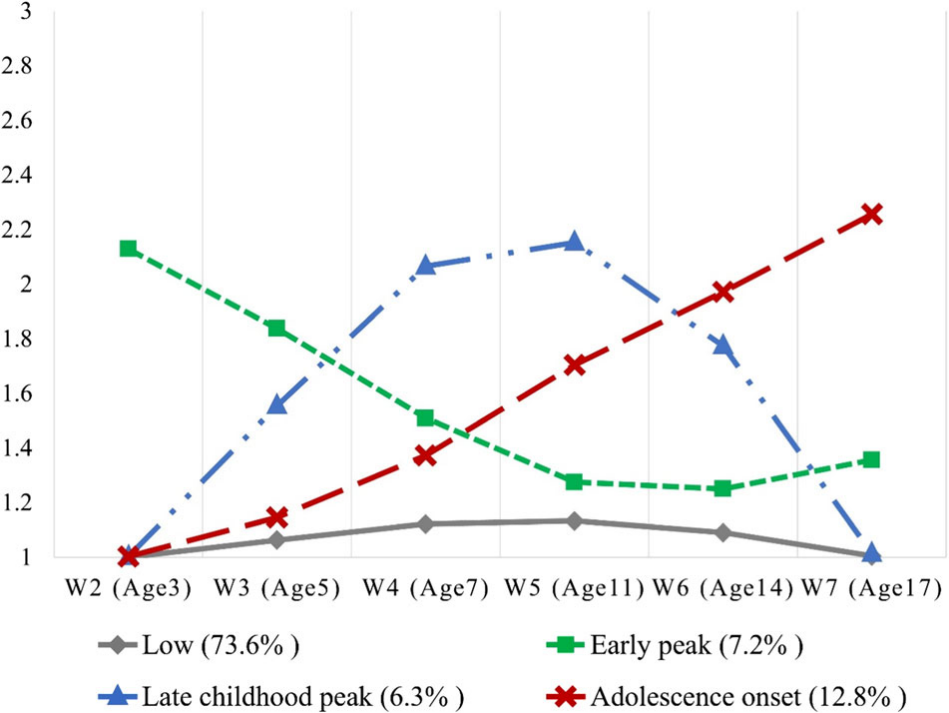
Victimization trajectories of the 4-class solution. Victimization was reported by parents with one item “Picked on or bullied by other children”, rating from 1 = *Not true* to 3 = *Very true*

A series of LCGMs with 1 to 5 classes was performed to identify the peer bullying victimization trajectories (see Table S11 for model fit). The AIC/aBIC kept leveling off when the number of classes increased, and the LMR-LRTs, aLRTs, and BLRTs were significant for all tested models, which indicated that the 5-class model was acceptable. However, the smallest class of the 5-class solution covered less than 5% of participants, which would limit the power to further test for SGD disparities considering the small sample size of SGD youth. Moreover, each trajectory identified in the 4-class solution (see Fig. 1) exhibited a unique pattern, ensuring that no class was merely a subset of another. Thus, the 4-class model was selected as the most parsimonious solution (see Table S12 for intercept, slope, and quadratic factors). Youth in Class 1 (73.6%) had a low risk of peer bullying victimization over time (Low Victimization Class); youth in Class 2 (7.2%) experienced the highest risk of victimization in early childhood, which subsequently decreased and remained low from late childhood onwards (Early Peak Victimization Class); youth in Class 3 (6.3%) showed a low risk in early childhood and late adolescence, with the risk peaking from middle to late childhood (Late Childhood Peak Victimization Class); and youth in Class 4 (12.8%) displayed heightened risk of victimization, which became especially visible since early adolescence (Adolescence Onset Victimization Class).

### SGD Status and Victimization Class Membership

The distributions of SGD youth among peer bullying victimization classes are presented in Table 1 and the 3-way crosstabulation of SO/GI subgroups and victimization trajectories is presented in Table S5. Table 2 presents the results testing the predictive roles of SGD identities on victimization class membership. The high entropy (0.92) suggested a high level of certainty in the classification and thereby validated the use of class membership as an indicator for latent trajectories.

**Table 1:** SGD youth’s distribution on most likely class membership based on the 4-class solution

Trajectory		Sexual Attraction			Sexual Identity			Gender Identity		SGD	non-SGD
		Sexual-attraction diverse	Mostly other-sex-attracted	Heterosexual	Sexual-identity diverse	Mainly heterosexual	Heterosexual	Gender diverse	Cisgender		
Overall valid sample size and percentage		1015	1378	7637	1076	1098	7868	109	9913	2722	7312
		10.07%	13.67%	75.76%	10.67%	10.89%	78.06%	1.08%	98.34%	27.00%	72.54%
Low	Sample size	628	1026	5866	672	835	6020	62	7452	1899	5625
	Percentage	61.87%	74.46%	76.81%	62.45%	76.05%	76.51%	56.88%	75.17%	69.76%	76.93%
Early Peak	Sample size	99	82	508	84	64	543	8	682	199	491
	Percentage	9.75%	5.95%	6.65%	7.81%	5.83%	6.90%	7.34%	6.88%	7.31%	6.71%
Adolescence Onset	Sample size	212	192	831	235	142	861	35	1197	451	783
	Percentage	20.89%	13.93%	10.88%	21.84%	12.93%	10.94%	32.11%	12.08%	16.57%	10.71%
Late Childhood Peak	Sample size	76	78	432	85	57	444	4	582	173	413
	Percentage	7.49%	5.66%	5.66%	7.90%	5.19%	5.64%	3.67%	5.87%	6.36%	5.65%

The SGD subgroup in this table includes *sexual-attraction diverse*, *sexual-identity diverse*, *mainly heterosexual, mostly other-sex-attracted*, and *gender diverse* youth. Percentages in this table present the distribution within each SGD-subsample (each column adds up to 100%)

*SGD* Sexually and Gender Diverse

**Table 2:** The predictive roles of SGD status on victimization class membership

SO/GI	Reference class	SGD status	Membership Class	Est.	OR	95% CI
Sexual attraction	Low	SA_D	Early peak	0.63***	1.88	1.49, 2.38
		SA_M		0.09	1.09	0.85, 1.40
		SA_D	Late childhood Peak	0.58***	1.78	1.37, 2.32
		SA_M		0.23	1.25	0.97, 1.62
		SA_D	Adolescence onset	0.84***	2.32	1.94, 2.77
		SA_M		0.30**	1.35	1.14, 1.61
	Adolescence onset	SA_D	Early peak	−0.21	0.81	0.62, 1.06
		SA_M		−0.22	0.81	0.61, 1.08
		SA_D	Late childhood Peak	−0.27	0.77	0.57, 1.03
		SA_M		−0.08	0.93	0.69, 1.24
	Late childhood peak	SA_D	Early peak	0.05	1.06	0.76, 1.47
		SA_M		−0.14	0.87	0.62, 1.23
Sexual identity	Low	SI_D	Early peak	0.45***	1.56	1.22, 2.00
		SI_M		0	1.00	0.76, 1.31
		SI_D	Late childhood Peak	0.66***	1.94	1.50, 2.49
		SI_M		0.11	1.11	0.83, 1.48
		SI_D	Adolescence onset	0.87***	2.39	2.02, 2.84
		SI_M		0.23*	1.26	1.04, 1.53
	Adolescence onset	SI_D	Early peak	−0.43**	0.65	0.50, 0.86
		SI_M		−0.24	0.79	0.58, 1.09
		SI_D	Late childhood Peak	−0.21	0.81	0.61, 1.07
		SI_M		−0.13	0.88	0.63, 1.22
	Late childhood peak	SI_D	Early peak	−0.22	0.81	0.58, 1.12
		SI_M		−0.11	0.90	0.61, 1.32
Gender identity	Low	GD	Early peak	0.47^a^	1.60	0.77, 3.32
		GD	Late childhood peak	−0.08^a^	0.92	0.33, 2.58
		GD	Adolescence onset	1.23***	3.40	2.25, 5.16

The predictive roles of SO/GIs are tested compared to heterosexual and cisgender youth;

*SGD* Sexually and Gender Diverse, *SO/GI* Sexual Orientation and Gender Identity, *SA_D* Sexual-attraction Diverse, *SA_M* Mostly Other-sex-attracted, *SI_D* Sexual-identity Diverse, *SI_M* Mainly Heterosexual, *GD* Gender Diverse

*p < 0.05, **p < 0.01, ***p < 0.001

^a^Due to the low numbers of gender diverse youth in the Early peak and Late childhood peak victimization classes, it is important to exercise caution when interpreting these results

Regarding sexual orientation, when using the Low Victimization class as the reference class, both youth who were *sexual-attraction diverse* and *sexual-identity diverse* showed a significantly higher risk of being classified in the three victimization-involved classes. Those identifying as *mainly heterosexual* or *mostly other-sex-attracted* were only at higher risk of being classified in the Adolescence Onset Victimization Class than in the Low Victimization class. Moreover, when the Adolescence Onset Victimization Class was set as the reference class, *sexual-identity diverse* youth showed decreased odds of being classified in the Early Peak Victimization Class.

Regarding gender identity, given the low numbers of *gender diverse* youth in the Early Peak (*n* = 8) and Late Childhood Peak (*n* = 4) classes, results related to these two classes should be interpreted with caution, and only the Low Victimization class was conducted as the reference class. Findings showed that *gender diverse* youth were more likely than cisgender youth to be classified in the Adolescence Onset Victimization Class.

### Mental Health Factors Associated with SGD Youth’s Victimization Trajectories

When significant main effects (SGD status on victimization class membership) were found, the indirect effects of SDG status via mental health and relational factors on victimization trajectory memberships were further tested. Unfortunately, the sample size of gender diverse youth was too small to test factors related to their victimization trajectories.

Results of the indirect effects via mental health factors are reported in Table 3 and Table S13. Some results are reported in the supplemental materials: (1) The results related to self-esteem (Table S13), which displayed similar roles as other mental health symptoms in SGD youth’s victimization trajectories; (2) the main effects (from SGD to the mediators and from the mediators to victimization) of the indirect models (Appendix J).

**Table 3:** Indirect effects with mental health factors as mediators

Mediator	Class membership		Sexual orientation	Wave 2		Wave 3		Wave 4		Wave 5		Wave 6	
	Reference	Outcome		b	95% CI	b	95% CI	b	95% CI	b	95% CI	b	95% CI
Emotional problems (Parent-reported)	Low	EP	SA_D	0.01	−0.04, 0.06	0.05*	0.01, 0.09	0.09*	0.04, 0.13	0.11*	0.07, 0.15	0.15*	0.11, 0.20
		LCP		0.01	−0.02, 0.03	0.04*	0.01, 0.07	0.10*	0.05, 0.15	0.18*	0.12, 0.23	0.23*	0.17, 0.29
		AO		0	−0.01, 0.01	0.02*	0, 0.04	0.06*	0.03, 0.09	0.16*	0.11, 0.21	0.26*	0.20, 0.32
		AO	SA_M	−0.01	−0.02, 0	−0.01	−0.02, 0.01	0.01	−0.01, 0.03	0.04	0, 0.08	0.06*	0.02, 0.11
		EP	SI_D	0.02	−0.03, 0.06	0.02	−0.02, 0.05	0.06*	0.02, 0.10	0.11*	0.07, 0.15	0.14*	0.10, 0.18
		LCP		0.01	−0.02, 0.03	0.01	−0.02, 0.04	0.07*	0.03, 0.12	0.18*	0.12, 0.23	0.21*	0.15, 0.26
		AO		0	−0.01, 0.01	0.01	−0.01, 0.03	0.05*	0.02, 0.08	0.16*	0.11, 0.21	0.24*	0.18, 0.30
		AO	SI_M	−0.01	−0.02, 0	−0.01	−0.02, 0.01	0.01	−0.02, 0.04	0.04	0, 0.08	0.06	0.01, 0.11
	AO	EP	SI_D	0.01	−0.03, 0.05	0.01	−0.01, 0.03	0.02*	0, 0.03	−0.05*	−0.07, −0.02	−0.10*	−0.14, −0.06
Emotional problems (Youth-reported)	Low	EP	SA_D	-	-	-	-	0	−0.01, 0.01	0.03*	0.01, 0.05	0.05*	0.01, 0.09
		LCP		-	-	-	-	0.02*	0, 0.03	0.09*	0.06, 0.12	0.11*	0.07, 0.15
		AO		-	-	-	-	0	0, 0.01	0.09*	0.06, 0.12	0.17*	0.13, 0.22
		AO	SA_M	-	-	-	-	0.01	−0.01, 0.02	0.07*	0.05, 0.10	0.17*	0.14, 0.21
		EP	SI_D	-	-	-	-	0	−0.01, 0.02	0.03*	0.01, 0.06	0.06*	0.01, 0.11
		LCP		-	-	-	-	0.03*	0.01, 0.05	0.10*	0.06, 0.13	0.13*	0.08, 0.19
		AO		-	-	-	-	0.01	−0.01, 0.02	0.10*	0.07, 0.13	0.21*	0.17, 0.26
		AO	SI_M	-	-	-	-	0	−0.01, 0.01	0.07*	0.04, 0.09	0.12*	0.09, 0.15
	AO	EP	SI_D	-	-	-	-	0	−0.02, 0.01	-0.06*	−0.09, −0.03	−0.15*	−0.21, −0.09
Hyperactive/inattention problems	Low	EP	SA_D	0.03*	0.01, 0.05	0.05*	0.03, 0.08	0.06*	0.04, 0.09	0.07*	0.04, 0.09	0.04*	0.01, 0.06
		LCP		0.03*	0.01, 0.05	0.07*	0.04, 0.10	0.11*	0.07, 0.14	0.11*	0.07, 0.15	0.05*	0.02, 0.08
		AO		0.02*	0.01, 0.04	0.06*	0.03, 0.08	0.10*	0.06, 0.13	0.11*	0.07, 0.15	0.06*	0.02, 0.10
		AO	SA_M	−0.02	−0.03, 0	0.01	−0.02, 0.03	0.02	−0.01, 0.04	0.03	0, 0.06	0.01	−0.02, 0.04
		EP	SI_D	0.02	−0.01, 0.04	0.04*	0.01, 0.06	0.05*	0.03, 0.08	0.07*	0.04, 0.10	0.04*	0.02, 0.06
		LCP		0.02	−0.01, 0.04	0.05*	0.02, 0.08	0.09*	0.05, 0.12	0.11*	0.07, 0.15	0.06*	0.02, 0.09
		AO		0.01	−0.01, 0.03	0.04*	0.01, 0.06	0.08*	0.05, 0.11	0.11*	0.07, 0.15	0.07*	0.03, 0.11
		AO	SI_M	−0.02	−0.04, 0	−0.01	−0.03, 0.01	−0.01	−0.04, 0.01	−0.01	−0.04, 0.02	−0.02	−0.06, 0.01
	AO	EP	SI_D	0	0, 0.01	0	−0.01, 0.01	−0.03*	−0.05, −0.01	−0.04*	−0.07, −0.02	−0.03*	−0.05, −0.01

*SA_D* Sexual-attraction Diverse, *SA_M* Mostly Other-sex-attracted, *SI_D* Sexual-identity Diverse, *SI_M* Mainly Heterosexual, *Low* Low Victimization Class, *EP* Early Peak Victimization Class, *LCP* Late Childhood Peak Victimization Class, *AO* Adolescence Onset Victimization Class

*Significant results after correcting for α level for multiple testing

Regarding mental health, the indirect effects were found for being sexually diverse (compared to exclusively heterosexual) on membership of the three victimized classes (compared to the Low Victimization class) via higher levels of emotional and hyperactive/inattention problems, and lower self-esteem. In terms of age, the indirect effect via hyperactive/inattention problems was observed since age 3 (W2) for *sexual-attraction diverse* youth and age 5 (W3) for *sexual-identity diverse* youth, while the indirect effect via emotional problems was observed since age 5 (W3) for *sexual-attraction diverse* youth and age 7 (W4) for *sexual-identity diverse* youth. In addition, indirect effects were found for youth who identified as *mainly heterosexual* or *mostly other-sex-attracted* (compared to exclusively heterosexual) on membership of the Adolescence Onset Victimization Class (compared to the Low Victimization class) via higher levels of emotional problems and lower self-esteem. Hyperactivity/inattention problems did not show indirect effects.

When using the Adolescence Onset Victimization Class as the reference class, the risk of *sexual-identity diverse* youth being in this class compared to the Early Peak Victimization Class was mediated by more emotional problems starting from age 11 (W5) and more hyperactive/inattention problems starting from age 7 (W4). Lower self-esteem also showed a mediating role for their victimization, but only at age 11 (W5), not at age 14 (W6).

### Relational Factors Associated with SGD Youth’s Victimization Trajectories

The indirect effects via relational factors are presented in Table 4 and Table S13. The indirect results via peer exclusion, social disclosure, and social support are reported in the appendix (Table S13), and the main effects of relational factors are reported in Appendix J. When using the Low Victimization class as the reference class, results showed that higher levels of peer problems, fewer available friendships, lack of parent-child closeness, and less available social support experienced by *sexually diverse* youth mediated their higher likelihood of membership of the three victimization-involved trajectories. In terms of age, the indirect effects via more peer problems were observed since age 3. Due to a lack of data on previous waves, the onset of other interpersonal factors was not clear. Notably, the significant indirect role of parent-child closeness was only found for youth-reported data but not for parent-reported ones.

**Table 4:** Indirect effects with relational factors as mediators

Mediator	Class membership		Sexual orientation	Wave 2		Wave 3		Wave 4		Wave 5		Wave 6	
	Reference	Outcome		b	95% CI	b	95% CI	b	95% CI	b	95% CI	b	95% CI
Peer problems (Parent-reported)	Low	EP	SA_D	0.09*	0.05, 0.14	0.12*	0.08, 0.16	0.16*	0.12, 0.21	0.22*	0.17, 0.28	0.26*	0.20, 0.31
		LCP		0.04*	0.02, 0.07	0.11*	0.07, 0.14	0.22*	0.17, 0.28	0.34*	0.27, 0.40	0.36*	0.29, 0.43
		AO		0.03*	0.01, 0.04	0.07*	0.04, 0.10	0.16*	0.12, 0.20	0.32*	0.26, 0.38	0.43*	0.36, 0.51
		AO	SA_M	0	−0.01, 0.01	0.02*	0, 0.03	0.04*	0.02, 0.07	0.10*	0.05, 0.14	0.16*	0.11, 0.21
		EP	SI_D	0.08*	0.03, 0.12	0.10*	0.06, 0.13	0.15*	0.11, 0.20	0.22*	0.17, 0.27	0.25*	0.20, 0.31
		LCP		0.04*	0.01, 0.06	0.09*	0.05, 0.12	0.21*	0.16, 0.26	0.31*	0.25, 0.38	0.35*	0.28, 0.42
		AO		0.02*	0.01, 0.04	0.06*	0.04, 0.08	0.15*	0.11, 0.19	0.30*	0.24, 0.36	0.42*	0.35, 0.49
		AO	SI_M	0.01	−0.01, 0.02	0.02*	0, 0.04	0.03*	0, 0.06	0.08*	0.03, 0.13	0.17*	0.12, 0.23
	AO	EP	SI_D	0.06*	0.02, 0.09	0.04*	0.01, 0.06	0.01	−0.02, 0.04	−0.09*	−0.13, −0.04	−0.17*	−0.23, −0.11
Availability of friendships (Youth-reported)	Low	EP	SA_D	-	-	-	-	0.01	0, 0.03	0.02	0, 0.03	0.02	−0.02, 0.05
		LCP		-	-	-	-	0.03*	0.01, 0.05	0.02*	0, 0.04	0.02	−0.02, 0.06
		AO		-	-	-	-	0.02*	0, 0.03	0.03*	0.01, 0.05	0.11*	0.08, 0.14
		AO	SA_M	-	-	-	-	0.02*	0.01, 0.03	0.03*	0.02, 0.05	0.03*	0.01, 0.05
		EP	SI_D	-	-	-	-	0.01	0, 0.02	0.02*	0.01, 0.04	0.02	−0.01, 0.04
		LCP		-	-	-	-	0.03*	0.01, 0.04	0.03*	0.01, 0.05	0.01	−0.01, 0.04
		AO		-	-	-	-	0.01*	0, 0.03	0.04*	0.02, 0.05	0.08*	0.06, 0.11
		AO	SI_M	-	-	-	-	0.02*	0, 0.03	0.03*	0.01, 0.05	0.05*	0.03, 0.07
	AO	EP	SI_D	-	-	-	-	0	−0.02, 0.01	−0.01	−0.03, 0.01	−0.07*	−0.10, −0.03

Mediator	Reference	Outcome	Sexual orientation	Wave 3 (Parent-reported)		Wave 4 (Parent-reported)		Wave 5 (Parent-reported)		Wave 6 (Parent-reported)		Wave 6 (Youth-reported)	
Parent-child closeness	Low	EP	SA_D	0.01	−0.01, 0.02	0.01	−0.01, 0.02	0	0, 0.01	0.01	0, 0.02	0.03*	0.01, 0.05
		LCP		0	−0.01, 0	0	0, 0.01	0	0, 0.01	0	0, 0.01	0.03*	0.01, 0.05
		AO		0	0, 0.01	0	0, 0.01	0	0, 0.01	0.01	0, 0.01	0.04*	0.02, 0.06
		AO	SA_M	0	0, 0	0	0, 0	0	−0.01, 0	0.01	0, 0.02	0.05*	0.03, 0.07
		EP	SI_D	0.01	0, 0.02	0	−0.01, 0.01	0	0, 0.01	0.01	0, 0.02	0.05*	0.02, 0.07
		LCP		0	−0.01, 0	0	−0.01, 0.01	0	0, 0.01	0	0, 0.01	0.04*	0.01, 0.07
		AO		0	0, 0.01	0	0, 0	0	0, 0.01	0.01	0, 0.01	0.06*	0.03, 0.08
		AO	SI_M	0	0, 0	0	0, 0	0	−0.01, 0	0.01	0, 0.02	0.03*	0.02, 0.05
	AO	EP	SI_D	0.01	0, 0.02	0	−0.01, 0.01	0	−0.01, 0	0.01	0, 0.02	−0.01	−0.04, 0.02

*SA_D* Sexual-attraction Diverse, *SA_M* Mostly Other-sex-attracted, *SI_D* Sexual-identity Diverse, *SI_M* Mainly Heterosexual, *Low* Low Victimization Class, *EP* Early Peak Victimization Class, *LCP* Late Childhood Peak Victimization Class, *AO* Adolescence Onset Victimization Class

*Significant results after correcting for α level for multiple testing

Similar indirect effects via relational factors were found for youth who identified as *mainly heterosexual* or *mostly other-sex-attracted*. However, peer exclusion observed at age 7 (W4) also significantly mediated the effect of these youth on the membership of the Adolescence Onset but not Low Victimization Class.

When using the Adolescence Onset Victimization Class as the reference class, less available friendships for *sexual-identity diverse* youth were found to mediate their decreased odds of being in the Early Peak Victimization Class (compared to heterosexual youth). A mediating role of peer problems was also found, but it should be noted that bullying victimization and peer problems were measured with items from the same subscale, and thus, they might be likely to co-develop.

### The Role of SGD Youth’s Victimization Trajectories in Late Adolescent Health and Well-Being

The posterior probabilities of the 3 victimization-involved trajectories were used as mediators of the effects of SGD status on late adolescent health and well-being. Table 5 reports the main effects of SGD status on health and well-being, and the indirect effects via victimization trajectories. The effects of SGD status on victimization, and, of victimization on well-being factors are reported in Appendix K.

**Table 5:** Late adolescent health and well-being related to SGD youth’s victimization trajectories

Health & well-being factors		SGD status	SGD → Health/Well-being		Indirect effect (via posterior probabilities)					
					Early peak		Adolescence onset		Late childhood peak	
			*β*	95% CI	b	95% CI	b	95% CI	b	95% CI
Self-harm		SA_D	0.21***	0.19, 0.23	0*	0.00, 0.01	0.04***	0.03, 0.04	0*	0, 0.01
		SA_M	0.19***	0.17, 0.21	0	0, 0	0.01**	0, 0.02	0*	0, 0.01
		SI_D	0.28***	0.27, 0.30	0	0, 0	0.04***	0.03, 0.04	0*	0, 0.01
		SI_M	0.11***	0.10, 0.13	0	0, 0	0.01	0, 0.01	0	0, 0
		GD	0.14***	0.12, 0.16	0	−0.01, 0.01	0.07***	0.05, 0.10	0	−0.01, 0.01
Emotional problems		SA_D	0.18***	0.16, 0.20	0*	0, 0.01	0.03***	0.02, 0.04	0**	0, 0.01
		SA_M	0.22***	0.20, 0.23	0	0, 0	0.01**	0, 0.02	0*	0, 0.01
		SI_D	0.23***	0.22, 0.25	0	0, 0	0.03***	0.02, 0.04	0.01**	0, 0.01
		SI_M	0.16***	0.14, 0.18	0	0, 0	0.01	0, 0.01	0	0, 0
		GD	0.1***	0.08, 0.12	0	−0.01, 0.01	0.07***	0.04, 0.09	0	−0.01, 0.01
Health	General health	SA_D	−0.07***	−0.09, −0.06	0*	−0.01, 0	−0.03***	−0.04, −0.02	−0.01**	−0.01, 0
		SA_M	−0.09***	−0.11, −0.07	0	0, 0	−0.01	−0.01, 0	0	−0.01, 0
		SI_D	−0.11***	−0.13, −0.09	0	0, 0	−0.03***	−0.04, −0.03	−0.01**	−0.01, 0
		SI_M	−0.05***	−0.07, −0.03	0	0, 0	0	−0.01, 0	0	0, 0
		GD	−0.04***	−0.05, −0.02	0	−0.01, 0	−0.07***	−0.09, −0.05	0	−0.01, 0.01
	Social & behavioral health concerns	SA_D	0.15***	0.12, 0.19	0	−0.01, 0.01	0.08***	0.06, 0.10	0.01*	0, 0.01
		SA_M	0.07**	0.02, 0.12	0	0, 0	0.01	0, 0.03	0	0, 0.01
		SI_D	0.16***	0.12, 0.19	0	0, 0	0.09***	0.07, 0.11	0.01*	0, 0.01
		SI_M	0.02	−0.03, 0.07	0	0, 0	0.01	−0.01, 0.02	0	0, 0.01
		GD	0.08***	0.05, 0.11	0	0, 0	0.17***	0.13, 0.22	0	−0.02, 0.01
Substance use	Smoking	SA_D	0	−0.02, 0.02	0	0, 0	0*	0, 0	0*	0, 0
		SA_M	0.03*	0.01, 0.05	0	0, 0	0	0, 0	0*	0, 0
		SI_D	0.03*	0.01, 0.05	0	0, 0	0	0, 0	0**	0, 0
		SI_M	−0.01	−0.03, 0.01	0	0, 0	0	0, 0	0	0, 0
		GD	0.01	−0.01, 0.03	0	0, 0	0	0, 0.01	0	0, 0
	Vaping	SA_D	0.01	−0.01, 0.03	0	0, 0	0**	0, 0	0	0, 0
		SA_M	0.03**	0.01, 0.05	0	0, 0	0*	0, 0	0	0, 0
		SI_D	0.03*	0.01, 0.05	0	0, 0	0**	0, 0	0	0, 0
		SI_M	0.01	−0.01, 0.03	0	0, 0	0	0, 0	0	0, 0
		GD	0.01	−0.01, 0.03	0	0, 0	0**	0, 0.01	0	0, 0
	Alcohol	SA_D	−0.03**	−0.05, −0.01	0	0, 0	0**	−0.01, 0	0*	0, 0
		SA_M	0.03**	0.01, 0.05	0	0, 0	0*	0, 0	0	0, 0
		SI_D	−0.02	−0.04, 0	0	0, 0	0**	−0.01, 0	0*	0, 0
		SI_M	0.02	0, 0.04	0	0, 0	0	0, 0	0	0, 0
		GD	−0.01	−0.03, 0.01	0	0, 0	−0.01**	−0.01, 0	0	0, 0
	Cannabis	SA_D	0.03*	0.01, 0.05	0	0, 0	−0.01***	−0.01, 0	0	0, 0
		SA_M	0.11***	0.09, 0.13	0	0, 0	0*	0, 0	0	0, 0
		SI_D	0.06***	0.04, 0.08	0	0, 0	−0.01***	−0.01, 0	0	0, 0
		SI_M	0.06***	0.04, 0.08	0	0, 0	0	0, 0	0	0, 0
		GD	0	−0.02, 0.02	0	0, 0	−0.01**	−0.01, 0	0	0, 0

In this table, the main effect estimates are presented as standardized coefficients (*β*), while the indirect effect estimates are reported as unstandardized coefficients (b)

*SGD* Sexually and Gender Diverse, *SA_D* Sexual-attraction Diverse, *SA_M* Mostly Other-sex-attracted, *SI_M* Sexual-identity Diverse, *SI_M* Mainly Heterosexual, *GD* Gender Diverse

*p < 0.05, **p < 0.01, ***p < 0.001

Indirect effects of *sexually diverse* youth compared to heterosexual youth via the probabilities of belonging to victimization classes were found for more self-harm behaviors, more emotional problems, poorer general health, and more mental and social health concerns. These compromised health and behavioral challenges faced by sexually diverse youth were mostly mediated by their Late Childhood Peak Victimization and Adolescence Onset Victimization patterns. Youth who identified as *mostly other-sex-attracted* also had more self-harm behaviors and emotional problems, which were mediated by the probabilities of Late Childhood Peak Victimization and Adolescence Onset Victimization Classes.

Compared to cisgender youth, significant indirect effects via Adolescence Onset Victimization were found for *gender diverse* youth on self-harm behaviors, emotional problems, general health, and mental and social health concerns.

As to substance use, while youth who were *sexual-identity diverse* and *mostly other-sex-attracted* reported higher levels of smoking and vaping, the indirect effects via victimization trajectories were very small (lower than 0.005). Also, although several indirect effects were found of SGD status on alcohol and cannabis use, these results were counter-intuitive: *Sexual-attraction diverse* youth reported less alcohol use than heterosexual youth, and higher probabilities of belonging to the victimized classes were significantly negatively associated with higher levels of alcohol and cannabis use. Thus, victimization negatively mediated some SGD youth’s alcohol and cannabis use.

### Sensitivity Analysis

The following sensitivity analyses were conducted in the current study. First, the pre-registered auxiliary approach was applied to test the predictive role of SGD status on peer bullying victimization trajectories (see Table S21). As shown, results were highly consistent between the pre-registered auxiliary method and the final-used class membership method, and thus, using the class membership method did not change the main conclusions of this study. Second, sensitivity analyses were conducted for all the main analyses, in which all the different SGD subgroups were merged into one general SGD group (see Appendix M). Results were highly consistent with the reported main analyses. Third, the consistency between youth’s self-reported victimization and parent-reported children’s peer bullying victimization was tested. The youth-report measures and the cross-informants consistency are reported in Appendix N. Results indicated that parents tended to under-report their children’s victimization instead of falsely detecting it.

## Discussion

While research has mainly documented bullying victimization disparities between SGD and heterosexual, cisgender youth from adolescence onwards, these disparities’ early development, associated vulnerabilities, and their roles in late adolescent well-being remained unclear. The current study expanded prior knowledge of peer bullying victimization disparities for SGD youth by examining these disparities from early childhood to late adolescence, exploring the mental health and relational factors associated with these disparities reported by both youth and parents, as well as testing late adolescent health and well-being outcomes related to SGD youth’s peer bullying victimization. Using a person-centered perspective, four trajectories of victimization were identified, including an early peak, late childhood peak, adolescence onset, and low victimization class. Results showed four important findings. First, in comparison with heterosexual, cisgender youth, SGD youth, in general, were at a higher risk of being in the victimization-involved classes, especially the adolescence onset victimization class. Second, youth in different SGD subgroups showed different probabilities belonging to different victimization trajectories. While *sexually diverse* youth showed heightened risks of being in all victimized classes, youth who identified as *gender diverse*, *mainly heterosexual*, or *mostly other-sex-attracted* showed a specific risk of being victimized from adolescence onwards. Third, the discrepancies in several mental health and relational problems between SGD and heterosexual, cisgender youth emerged before late childhood, and such factors were associated with the risk of SGD youth’s victimization trajectories. Fourth, results showed that SGD youth experienced more emotional problems, more self-harm behaviors, and worse health conditions in late adolescence, which could be partially explained by their risk of peer bullying victimization through childhood and adolescence.

### SGD Disparities in Victimization Trajectories

First, findings partly supported the hypothesis that SGD youth would be more persistently victimized in childhood and adolescence than heterosexual, cisgender youth, and the victimization among SGD youth might already start in childhood presenting long-term exposure patterns. While previous evidence showed that SGD youth were more persistently victimized during adolescence (Kaufman et al., 2020), the current study extended previous research into childhood, and found clues of long-term victimization among SGD children. While *sexually diverse* youth were more likely to be in all three victimized trajectories, SGD youth, in general, displayed a higher risk of being victimized from adolescence onwards compared to heterosexual cisgender youth. This is interesting because youth with an adolescence onset pattern displayed a conspicuous risk of bullying victimization in middle and late adolescence, while all other youth tended to have a low or decreasing probability of victimization. The general level of peer bullying victimization would be expected to decline during adolescence, which might be due to the development of cognitive skills and the ability to avoid bullies (Troop-Gordon, 2017, for a review). However, for SGD youth, adolescence is a milestone period in which they have more diverse sexual experiences, explore their diverse identities, and might also disclose their identities (Mustanski et al., 2014). Also, adolescence is a particularly vulnerable period during which SGD youth experience a developmental collision (Russell & Fish, 2019). That is, with growing social acceptance, SGD youth tend to disclose their identity earlier in adolescence, which, however, exposes them to the risk of victimization at an age with limited supportive or protective social, interpersonal, and individual resources. Thus, during adolescence, SGD youth would become more visible in a cisgender, heteronormative peer context, which could aggravate their risk of victimization. Altogether, the findings emphasize the seriousness of SGD youth’s victimization because their increased risk in adolescence might outweigh their gained ability to fight against victimization.

In addition, regarding intragroup differences among SGD youth, *sexually diverse* youth (both by attraction and identity) displayed higher risks of all victimization patterns, while youth who were *mainly heterosexual* and *mostly other-sex-attracted* only showed a risk of adolescence onset victimization. That is, victimization among *sexually diverse* youth could be a more complex issue characterized by mixed risks. A possible reason is that sexual-related behaviors among *sexually diverse* youth deviate more from heteronormativity than youth who are *mainly heterosexual* and *mostly other-sex-attracted* in terms of identity or expression (Thompson & Morgan, 2008), which makes them more visible among peers and more easily targeted by perpetrators.

### Associated Factors of Disparities in SGD Youth’s Bullying Victimization Trajectories

In line with the hypotheses, several mental health and relational factors were found related to the trajectories of SGD youth’s peer bullying victimization. First, support was found for all proposed mental health factors related to the disparities in SGD youth’s victimization trajectories, and some of the links already appeared in childhood. The risk of long-term bullying victimization exposure for *sexually diverse* youth, compared to heterosexual youth, was related to more hyperactivity/inattention, more emotional problems, and lower self-esteem. These psychological symptoms were related to the persistence of victimization, perhaps because youth with these issues are more likely to be targeted by bullies and less likely to have proper coping strategies after being victimized. Although a previous cohort study suggested that sexual orientation disparities in internalizing symptoms appeared at age 12 (Pachankis et al., 2022), the current results suggest that mental health disparities emerge before middle childhood. Before developing a mature sexual orientation, early clues of sexual diversity can appear around middle childhood (Mustanski et al., 2014), and may be related to the early onset of mental vulnerabilities for youth who later identified as SGD.

Second, most of the hypothesized associations of relational factors with SGD youth’s victimization trajectories were supported by the findings. To be specific, *sexually diverse* youth’s risk of long-term bullying victimization, compared to heterosexual youth, was related to a higher level of peer problems, lack of available friendships, lack of parent-child closeness, and less social support. In accordance with the generalized unsafety framework (Diamond & Alley, 2022), the difficulties that SGD youth face are not just the presence of minority stress, but also the absence of social safety, including sufficient social connection, inclusion, and protection, which is an important need for youth. Interpersonal relationships characterized by a lack of closeness, connection, and support could in turn lead to perceptions of unsafety among SGD youth, which could inhibit their help-seeking behaviors after being victimized (Diamond & Alley, 2022) and reduce their possibility of escaping from persistent victimization.

Notably, while findings drawn from both parent- and youth-reported information were mostly consistent, only youth self-reported but not parent-reported lack of parent-child closeness was associated with SGD youth’s victimization. Such perception bias can be explained from two different perspectives. First, parents might report more positive parent-child relationships than children (Hoeve et al., 2009). Thus, reports from SGD youth regarding a lack of parent-child closeness could be more effectively related to victimization disparities. Second, on the youth’s side, the identification of one’s diverse status might come along with the fear or expectation of parents’ rejection, which may lead to SGD youth perceiving less closeness with their parents, thus reflecting a bias in their perceptions. Meanwhile, the different perceptions of closeness between parents and SGD youth might also explain the divergence of parent- and youth- reported victimization found in the current study that parents of SGD youth were less likely to accurately detect their children’s victimization compared to parents of heterosexual, cisgender youth. When SGD youth perceive less closeness, they might share less about their lives with parents. In line, previous evidence indicated that SGD youth felt less safe to report victimization to their parents (Kaufman & Baams, 2022). However, this can in turn limit the support parents provide, which makes the improvement of SGD youth’s perceived closeness and support an important task to stop bullying and enhance their well-being.

In addition, most of the identified associated factors were consistent across sexual identity and attraction. Only the link between *sexually diverse* statuses and victimization via emotional and hyperactive/inattention problems could be observed earlier among *sexual-attraction diverse* youth than among *sexual-identity diverse* youth. This different timing found for sexual attraction and sexual identity might be related to their developmental features, with sexual attraction developing at a relatively younger age than sexual identity (Mustanski et al., 2014). This might make sexual attraction a more effective measure to identify disparities early in life. Although the overlap between sexual attraction and sexual identity found in the current sample was higher than in previous studies (Igartua et al., 2009), this finding supports the importance of distinguishing between sexual attraction and sexual identity.

### Late Adolescent Health and Well-Being Related to SGD Youth’s Bullying Victimization Trajectories

The findings that SGD youth reported more self-harm, emotional problems, and health concerns supported the minority stress framework (Meyer, 1995; 2003), which suggests that a minority identity brings SGD youth an elevated risk of victimization, especially longer-lasting peer bullying victimization, which then yields harm to their later health and well-being. Among all victimization patterns, being victimized from adolescence onwards, as a consistent risk faced by all SGD youth, was linked to most of the problematic behaviors and poor health conditions. This again emphasizes the seriousness of being trapped in longer-lasting bullying victimization while one is growing and maturing. However, results also showed that the trajectories of bullying victimization could only explain a small part of SGD youth’s well-being. This is understandable because, beyond peer bullying victimization, other potential stressors can yield significant harm to SGD youth’s development, such as other forms of minority stress (e.g., discrimination) (Meyer, 1995; 2003) and the lack of social safety (Diamond & Alley, 2022).

Additionally, an exceptional result was found for alcohol use: *sexually-attraction diverse* youth reported lower levels of alcohol use than heterosexual youth. Alcohol use is typically higher among more popular youth (e.g., Gommans et al., 2017), which might explain this finding. Moreover, although SGD youth reported more cannabis use, cannabis use was negatively associated with victimization probabilities. While cannabis use is considered problematic, it might sometimes be a sign of more connections with peers (e.g., Nelemans et al., 2016), because youth’s cannabis use is likely to happen in a peer context. Given that peer victimization presents a negative peer relationship, it is understandable to find a negative association between cannabis use and the risk of peer victimization.

### Strengths, Limitations, and Future Directions

This study provided novel insights into disparities in victimization trajectories between SGD and heterosexual, cisgender youth, extending the developmental perspective to early childhood. First, by adopting a person-centered approach, this study captured nuanced disparities beyond mean-level differences and explored individual differences within the victimized population. Second, the current study provided a more comprehensive view of victimization disparities by distinguishing the roles of sexual attraction and sexual identity, as well as between *sexually diverse* youth and those who were *mainly heterosexual/mostly other-sex attracted*. Third, using both parent- and youth-reported data, the findings revealed substantial consistency across informants regarding factors associated with SGD youth’s victimization trajectories. Fourth, the study also provided information about how the different developmental victimization patterns could explain late adolescent health and well-being discrepancies between SGD youth and heterosexual, cisgender youth.

However, this study also displayed some limitations. To begin with, the directionality between the mental health and relational factors and victimization remains unclear, and is likely to be bidirectional (van Geel et al., 2018). For example, while the findings indicated that emotional problems were associated with SGD youth’s victimization trajectories, and that SGD youth who had a higher risk of longer-lasting victimization trajectories exhibited more emotional problems in late adolescence, it is possible that emotional problems and persistent victimization co-developed over time. Although this study cannot clarify directionality, the findings emphasized that the presence of socioemotional vulnerabilities made it challenging for SGD youth to escape long-term victimization, and the potential vicious circle could also link to prolonged health difficulties. Further longitudinal studies are needed to clarify these relations.

Another limitation is that, while using parent-reported youth’s peer bullying victimization enabled the current study to trace the continuous development from early childhood, this measurement approach may introduce bias. Comparisons between parent- and youth-reported victimization indicated that parents could have underreported their children’s victimization, especially during late adolescence. However, even with such underreporting bias, a group of youth was identified as experiencing victimization from adolescence onwards, which was the risky victimization pattern shared by different SGD subgroups. This in turn emphasizes the severity of SGD youth’s persistent victimization during adolescence. Future research should explore the disparities between SGD and heterosexual cisgender youth using multi-informant data to reveal potential inconsistencies. Meanwhile, using single items to represent the general level of peer bullying victimization may overlook important details, such as specific forms and locations of victimization (Kaufman & Baams, 2022), which would be fruitful topics for future studies.

In addition, despite tracking victimization through childhood and adolescence, SO/GI items were solely reported in late adolescence. Youth may be aware of their SO/GI before late adolescence, with some youth expressing cross-gender identities in early childhood, reporting same-sex attractions during middle to late childhood, and identifying with a variety of non-heterosexual identity labels in middle to late adolescence (Bockting, 2014; Mustanski et al., 2014). Measuring age-appropriate experiences related to SO/GI at younger ages, such as early adolescence, is recommended but rarely done (Baams & Kaufman, 2023), and it would be insightful for future studies to explore.

Lastly, several sampling biases should be considered when interpreting the current findings. Youth who responded “prefer not to say” to the SO/GI items were excluded, which potentially resulted in an underestimation of disparities between SGD and heterosexual, cisgender youth. Also, the current sample lacked gender diversity, with only 1.03% of youth reporting gender identity not in line with their sex assigned at birth, which underscores the need for targeted sampling to study disparities in victimization trajectories among gender diverse youth. Moreover, while the MCS data aimed to be representative of the UK, extending these findings to other countries should be done cautiously due to potential differences in SGD social acceptance and policies.

## Conclusions

For scientific and intervention purposes aimed at enhancing the thriving of SGD youth, it is crucial to study the early development of peer bullying victimization, along with its associated vulnerabilities and relations with the well-being of SGD youth. This study examined the developmental bullying victimization disparities from early childhood to late adolescence, explored potential mental and relational factors that would be associated with the trajectories of victimization, and tested how the long-term exposure of peer bullying victimization would relate to SGD youth’s detrimental health and well-being during late adolescence. The current findings showed that peer bullying victimization disparities for SGD youth may already exist in childhood, and such disparities can be associated with their psychological and interpersonal difficulties from early childhood onward. Also, SGD youth, compared to heterosexual, cisgender youth, exhibited more behavioral challenges and poorer health in late adolescence, and long-term peer bullying victimization during childhood and adolescence may partly account for these discrepancies. Specifically, the adolescence-onset peer bullying victimization pattern is likely to persist throughout adolescence and constitutes a notably high risk when youth are transitioning into emerging adulthood, and as such could yield the most harm to SGD youth’s well-being. Altogether, the findings implied that to reduce SGD youth’s challenges during the transition to adulthood, early anti-bullying work should start before adolescence, and interventions to reduce their psychological and interpersonal burdens should already start in childhood. In sum, compared to heterosexual, cisgender youth, mental and relational vulnerabilities among SGD youth were established from childhood onwards and were associated with their heightened risk of experiencing longer-lasting peer bullying victimization through childhood and adolescence, which then linked to more health and behavioral challenges in late adolescence.

## Supplementary information

Supplementary Materials
