# Supplementary material for: Peer Bullying Victimization Trajectories for Sexually and Gender Diverse Youth from Early Childhood to Late Adolescence
Source: J Youth Adolesc. 2024 Jun 7;53(11):2589–609. doi: 10.1007/s10964-024-02020-8 (PMC11466994; doi:10.1007/s10964-024-02020-8)
Supplement: Supplementary file 1 — Supplementary Materials [file 10964_2024_2020_MOESM1_ESM.docx]

**Online Supplemental Materials for**

**Peer Bullying Victimization Trajectories for Sexually and Gender Diverse Youth from Early Childhood to Late Adolescence**

**Contents**

[Appendix A: Confirmatory Factor Analysis 3](#_Toc166241010)

[Appendix B: Deviations from Pre-Registration 5](#_Toc166241011)

[Appendix C: Figures of Analytical Models 7](#_Toc166241012)

[Appendix D: Distribution of Sexual Orientation, Gender Identity, and Victimization Trajectories 8](#_Toc166241013)

[Appendix E: Descriptive Analysis and Correlations 11](#_Toc166241014)

[Appendix F: Longitudinal Measurement Invariance 15](#_Toc166241015)

[Appendix G: Model Fit Indices for Latent Class Growth Models 16](#_Toc166241016)

[Appendix H: Intercept, Slope, and Quadratic Factors of the 4-Class Trajectory 17](#_Toc166241017)

[Appendix I: Indirect Effect of Associated Factors 18](#_Toc166241018)

[Appendix J: Main Effects of Associated Factors from SGD Status to Victimization Trajectories 19](#_Toc166241019)

[Appendix K: Main Effects of Victimization Trajectories from SGD Status to Late Adolescent Health and Well-Being 24](#_Toc166241020)

[Appendix L: The Pre-Registered Auxiliary Method 26](#_Toc166241021)

[Appendix M: Sensitivity Analyses Merging all SGD Subgroups 28](#_Toc166241022)

[Appendix N: Cross-Informant Comparisons Between Parent- and Youth-reported Victimization 38](#_Toc166241023)

# **Appendix A: Confirmatory Factor Analysis**

| Table S1 | | | | | | | | |
| --- | --- | --- | --- | --- | --- | --- | --- | --- |
| *Confirmatory Factor Analysis for Youth-Reported Emotional Problems* | | | | | | | | |
| Wave 4 | Model | Model fit | | | | |  |  |
|  |  | *χ*²/df | RMSEA | CFI | TLI | SRMR |  |  |
|  | 1 | 35.19321 | 0.062 | 0.863 | 0.794 | 0.041 |  |  |
|  | 2 | 39.92622 | 0.066 | 0.896 | 0.827 | 0.039 |  |  |
|  | 3 | 18.30540 | 0.044 | 0.970 | 0.941 | 0.022 |  |  |
|  | Model | Standardized factor loading | | | | | | |
|  |  | Happy | Worry | Sad | Quiet | Alone | Laugh | Lost temper |
|  | 1 | 0.49 | 0.43 | 0.61 | -0.03 | 0.34 | 0.22 | 0.43 |
|  | 2 | 0.48 | 0.43 | 0.61 | - | 0.34 | 0.22 | 0.43 |
|  | 3 | 0.44 | 0.45 | 0.63 | - | 0.33 | - | 0.45 |
| Wave 5 | Model | Model fit | | | | |  |  |
|  |  | *χ*²/df | RMSEA | CFI | TLI | SRMR |  |  |
|  | 1 | 118.9996 | 0.111 | 0.951 | 0.919 | 0.042 |  |  |
|  | 2 | 101.4736 | 0.103 | 0.976 | 0.953 | 0.025 |  |  |
|  | Model | Standardized factor loading | | | | | |  |
|  |  | Happy | Worry | Sad | Scared | Laugh | Angry |  |
|  | 1 | 0.50 | 0.70 | 0.78 | 0.77 | 0.17 | 0.61 |  |
|  | 2 | 0.49 | 0.70 | 0.78 | 0.77 | - | 0.61 |  |
| *Note.* On wave 4, Model 1 included all 7 original items, Model 2 excluded the *quiet* item, and Model 3 excluded both the *quiet* and *laugh* items. On wave 5, Model 1 included all 6 original items, and Model 2 excluded the *laugh* item. | | | | | | | | |

| Table S2 | | | | | | |
| --- | --- | --- | --- | --- | --- | --- |
| *Confirmatory Factor Analysis for Parent-Reported Emotional and Peer Problems* | | | | | | |
| Emotional problems (parent-reported) | Wave | Model fit | | | | |
|  |  | *χ*²/df | RMSEA | CFI | TLI | SRMR |
|  | 2 | 25.7900 | 0.053 | 0.967 | 0.934 | 0.038 |
|  | 3 | 17.3144 | 0.042 | 0.986 | 0.972 | 0.026 |
|  | 4 | 29.9532 | 0.056 | 0.981 | 0.962 | 0.028 |
|  | 5 | 62.4056 | 0.082 | 0.978 | 0.955 | 0.033 |
|  | 6 | 55.7124 | 0.078 | 0.982 | 0.963 | 0.029 |
|  | Wave | Standardized factor loading | | | | |
|  |  | Item 1 | Item 2 | Item 3 | Item 4 | Item 5 |
|  | 2 | 0.44 | 0.70 | 0.72 | 0.54 | 0.71 |
|  | 3 | 0.47 | 0.73 | 0.71 | 0.56 | 0.75 |
|  | 4 | 0.46 | 0.77 | 0.70 | 0.58 | 0.76 |
|  | 5 | 0.48 | 0.79 | 0.76 | 0.66 | 0.80 |
|  | 6 | 0.47 | 0.80 | 0.75 | 0.70 | 0.81 |
| Peer problems (parent-reported) | Wave | Model fit | | | | |
|  |  | *χ*²/df | RMSEA | CFI | TLI | SRMR |
|  | 2 | 6.941 | 0.026 | 0.997 | 0.984 | 0.008 |
|  | 3 | 1.240 | 0.005 | 1.000 | 0.999 | 0.004 |
|  | 4 | 2.865 | 0.014 | 0.999 | 0.996 | 0.006 |
|  | 5 | 2.756 | 0.014 | 1.000 | 0.997 | 0.005 |
|  | 6 | 0.609 | 0 | 1.000 | 1.001 | 0.002 |
|  | Wave | Standardized factor loading | | | | |
|  |  | Item 1 | Item 2 | Item 3 | Item 4 |  |
|  | 2 | 0.69 | 0.28 | 0.31 | 0.65 |  |
|  | 3 | 0.67 | 0.44 | 0.43 | 0.67 |  |
|  | 4 | 0.64 | 0.45 | 0.52 | 0.71 |  |
|  | 5 | 0.67 | 0.56 | 0.62 | 0.66 |  |
|  | 6 | 0.64 | 0.57 | 0.61 | 0.60 |  |

# **Appendix B: Deviations from Pre-Registration**

There were several deviations from the pre-registration (https://osf.io/f2zxy).

**1. Changes in measurement**

**a. Social and Behavioral Concerns:** In the pre-registration, we stated that social and behavioral concerns were measured with a single item “Do you have any socially or behaviourally conditions or illnesses lasting or expected to last 12 months or more”. However, upon closer examination of the dataset, we found a more general prior item asking “Do you have any physical or mental health conditions or illnesses lasting or expected to last 12 months or more?”, and only youth who responded yes to this question would further be asked about their socially or behaviourally health concerns. Thus, to include responses from those who answered '*no*' to this general question, we merged the two items in our final analysis for a more comprehensive analysis.

**b. Cannabis use:** In the pre-registration, we stated that social and behavioral concerns were measured with a single item “In the past year how many times have you taken Cannabis”. However, upon closer examination of the dataset, we found a prior item asking “Have you ever taken cannabis”, and only youth who responded yes would be asked to answer the frequency. Thus, to be inclusive of those who have never used cannabis, the two items were merged in our final analysis.

**2. Changes in analytical strategy**

a. In the pre-registration we planned to test the predictive roles of SGD statuses to victimization class membership (step 2) by assigning auxiliary variables, and we planned to test the related factors of persistent victimization (step 3) using the posterior probabilities as victimization indicators. However, in our final analysis, we used the most likely class membership as the indicator of victimization trajectories in these two steps. The exploratory nature of Latent Class Growth Models (LCGMs) prevented us from predicting the exact number and types of victimization trajectories beforehand. We identified 4 victimization trajectories and we did not find a high-stable class that can perfectly present persistent victimization. Instead, we found 3 victimized classes that all displayed a certain level of chronic exposure to victimization. Given this result, instead of using the posterior probabilities to address youth’s higher or lower chance of being persistently victimized, we want to explore the nuanced differences among the victimized classes. Therefore, we used the class membership as the indicator when testing the associated factors. Then, for consistency, we also used class membership as the indicator when exploring the predictive roles of SGD statuses in victimization trajectories (step 2). Consistent with what was reported in the pre-registration, we used the posterior probabilities as mediators when analyzing the health and well-being outcomes. This was due to the statistical difficulties of using a nominal variable (class membership) as a mediator. The reason for using only the probabilities of 3 classes was because all 4 probabilities added up to 1 for each participant, and thus, using all 4 probabilities simultaneously, the model won’t be able to converge due to redundant information.

# **Appendix C: Figures of Analytical Models**

SGD status

Associated factors:

- Emotional problems

- Hyperactive/inattention problems

- Self-esteem

- Peer problems

- Parent-child closeness

- Social support

Class memberships of victimization trajectories

**S1-a**

Posterior probabilities of trajectory 1

Health & Well-being:

- Emotional Problems

- Self-harm

- Health

- Substance use

**S1-b**

**Figure S1** Proposed models

SGD status

Posterior probabilities of trajectory 2

Posterior probabilities of trajectory *n*-1

…

# **Appendix D: Distribution of Sexual Orientation, Gender Identity, and Victimization Trajectories**

| Table S3 | | | | | | | | | |
| --- | --- | --- | --- | --- | --- | --- | --- | --- | --- |
| *Overlap Between Sexual Attraction and Sexual Identity* | | | | | | | | | |
| Sexual attraction | Sexual identity | | | | | | | | |
|  | Sexual-identity diverse  (*n* = 1076) | | | Mainly heterosexual  (*n* = 1098) | | | Exclusively heterosexual  (*n* = 7868) | | |
|  | *n* | Overlap%  (based on SI) | Overlap %  (based on SA) | *n* | Overlap %  (based on SI) | Overlap %  (based on SA) | *n* | Overlap %  (based on SI) | Overlap %  (based on SA) |
| Sexual-attraction diverse  (*n* = 1015) | 773 | 71.84% | 76.16% | 59 | 5.37% | 5.81% | 174 | 2.21% | 17.14% |
| Mostly other-sex attracted  (*n* = 1378) | 263 | 24.44% | 19.09% | 754 | 68.67% | 54.72% | 360 | 4.58% | 26.12% |
| Exclusively heterosexual  (*n* = 7637) | 36 | 3.35% | 0.47% | 283 | 25.77% | 3.71% | 7316 | 92.98% | 95.81% |
| *Note*. *SI* Sexual Identity, *SA* Sexual Attraction. | | | | | | | | | |

| Table S4 | | | |
| --- | --- | --- | --- |
| *Crosstabulation of Sexual Orientation and Gender Identity* | | | |
| Sexual orientation | | Gender identity | |
|  |  | Gender diverse (%) | Cisgender (%) |
| Sexual attraction | Sexual-attraction diverse | 85 (78.0%) | 900 (9.1%) |
|  | Mostly other-sex attracted | 12 (11.0%) | 1361 (13.7%) |
|  | Exclusively heterosexual | 11 (10.1%) | 7603 (76.7%) |
| Sexual identity | Sexual-identity diverse | 99 (90.8%) | 946 (9.5%) |
|  | Mainly heterosexual | 4 (3.7%) | 1085 (10.9%) |
|  | Exclusively heterosexual | 6 (5.5%) | 7847 (79.2%) |
| Sexual orientation | Non-heterosexual | 105 (96.3%) | 2596 (26.2%) |
|  | Exclusively heterosexual | 4 (3.7%) | 7318 (73.8%) |
| *Note*. The *non-heterosexual* orientation group includes *sexual-attraction diverse*, *sexual-identity diverse*, *mostly other-sex attracted*, and *mainly heterosexual* youth. | | | |

| Table S5 | | | | |
| --- | --- | --- | --- | --- |
| *Three-Way Crosstabulation of Sexual Orientation, Gender Identity, and Victimization Trajectories* | | | | |
| Victimization trajectory | Sexual orientation | Gender identity | | Total |
|  |  | Gender diverse | Cisgender |  |
| Low | Exclusively heterosexual | 4 | 5624 | 5628 |
|  | Non-heterosexual | 58 | 1808 | 1866 |
|  | Total | 62 | 7452 | 7514 |
| Early peak | Exclusively heterosexual | 0 | 493 | 493 |
|  | Non-heterosexual | 8 | 187 | 195 |
|  | Total | 8 | 682 | 690 |
| Adolescence onset | Exclusively heterosexual | 0 | 788 | 788 |
|  | Non-heterosexual | 35 | 406 | 441 |
|  | Total | 35 | 1197 | 1232 |
| Late childhood peak | Exclusively heterosexual | 0 | 413 | 413 |
|  | Non-heterosexual | 4 | 168 | 172 |
|  | Total | 4 | 582 | 586 |
| *Note.* The *non-heterosexual* orientation group includes *sexual-attraction diverse*, *sexual-identity diverse*, *mostly other-sex attracted*, and *mainly heterosexual* youth. | | | | |

# **Appendix E: Descriptive Analysis and Correlations**

| Table S6 | | | | | | | |
| --- | --- | --- | --- | --- | --- | --- | --- |
| *ANOVA for SGD Status and Victimization* | | | | | | | |
| SGD status | | F/*df* | | | | | |
|  |  | W2_Vic | W3_Vic | W4_Vic | W5_Vic | W6_Vic | W7_Vic |
| Sexual attraction | SA_D | 9.69** | 0.99 | 15.89*** | 83.75*** | 101.48*** | 97.34*** |
|  | SA_M | 0.96 | 2.41 | 0.03 | 7.63** | 2.89 | 3.59 |
| Sexual identity | SI_D | 0.61 | 0.03 | 16.45*** | 94.44*** | 102.75*** | 90.13*** |
|  | SI_M | 0.98 | 0.50 | 0.18 | 0.57 | 0.01 | 1.37 |
| Gender identity | GD | 0.07 | 0.33 | 0.10 | 15.68*** | 13.34*** | 47.76*** |
| *Note.* *SGD* Sexually and Gender Diverse, *SA_D* Sexual-attraction Diverse, *SA_M* Mostly Other-sex-attracted, *SI_D* Sexual-identity Diverse, *SI_M* Mainly Heterosexual, *GD* Gender Diverse, *W* Wave, *Vic* Victimization. Factorial ANOVA was conducted for sexual attraction and sexual identity, and normal ANOVA was conducted for gender identity.  **p* < .05, ***p* < .01, ****p* < .001. | | | | | | | |

| Table S7 | | | | | | | | | | | | | | | | | | | | | | | |
| --- | --- | --- | --- | --- | --- | --- | --- | --- | --- | --- | --- | --- | --- | --- | --- | --- | --- | --- | --- | --- | --- | --- | --- |
| *Correlations Between Victimization and Associated Mental Health Factors* | | | | | | | | | | | | | | | | | | | | | | | |
| Variable | | Mean | *SD* | 1 | 2 | 3 | 4 | 5 | 6 | 7 | 8 | 9 | 10 | 11 | 12 | 13 | 14 | 15 | 16 | 17 | 18 | 19 | 20 |
| 1 | W2_Vic | 1.09 | 0.32 |  |  |  |  |  |  |  |  |  |  |  |  |  |  |  |  |  |  |  |  |
| 2 | W3_Vic | 1.15 | 0.41 | 0.19 ^***^ |  |  |  |  |  |  |  |  |  |  |  |  |  |  |  |  |  |  |  |
| 3 | W4_Vic | 1.22 | 0.48 | 0.13 ^***^ | 0.28 ^***^ |  |  |  |  |  |  |  |  |  |  |  |  |  |  |  |  |  |  |
| 4 | W5_Vic | 1.28 | 0.54 | 0.1 ^***^ | 0.23 ^***^ | 0.31 ^***^ |  |  |  |  |  |  |  |  |  |  |  |  |  |  |  |  |  |
| 5 | W6_Vic | 1.28 | 0.56 | 0.08 ^***^ | 0.17 ^***^ | 0.23 ^***^ | 0.4 ^***^ |  |  |  |  |  |  |  |  |  |  |  |  |  |  |  |  |
| 6 | W7_Vic | 1.2 | 0.49 | 0.08 ^***^ | 0.13 ^***^ | 0.2 ^***^ | 0.31 ^***^ | 0.41 ^***^ |  |  |  |  |  |  |  |  |  |  |  |  |  |  |  |
| 7 | W2_Hyp | 1.75 | 0.47 | 0.1 ^***^ | 0.14 ^***^ | 0.14 ^***^ | 0.17 ^***^ | 0.15 ^***^ | 0.1 ^***^ |  |  |  |  |  |  |  |  |  |  |  |  |  |  |
| 8 | W3_Hyp | 1.63 | 0.47 | 0.1 ^***^ | 0.17 ^***^ | 0.18 ^***^ | 0.21 ^***^ | 0.18 ^***^ | 0.13 ^***^ | 0.57 ^***^ |  |  |  |  |  |  |  |  |  |  |  |  |  |
| 9 | W4_Hyp | 1.64 | 0.49 | 0.08 ^***^ | 0.14 ^***^ | 0.21 ^***^ | 0.24 ^***^ | 0.2 ^***^ | 0.15 ^***^ | 0.5 ^***^ | 0.67 ^***^ |  |  |  |  |  |  |  |  |  |  |  |  |
| 10 | W5_Hyp | 1.59 | 0.48 | 0.08 ^***^ | 0.14 ^***^ | 0.2 ^***^ | 0.3 ^***^ | 0.26 ^***^ | 0.18 ^***^ | 0.43 ^***^ | 0.56 ^***^ | 0.66 ^***^ |  |  |  |  |  |  |  |  |  |  |  |
| 11 | W6_Hyp | 1.57 | 0.47 | 0.08 ^***^ | 0.13 ^***^ | 0.18 ^***^ | 0.24 ^***^ | 0.27 ^***^ | 0.19 ^***^ | 0.39 ^***^ | 0.49 ^***^ | 0.56 ^***^ | 0.68 ^***^ |  |  |  |  |  |  |  |  |  |  |
| 12 | W2_Pemo | 0.03 | 0.3 | 0.26 ^***^ | 0.15 ^***^ | 0.11 ^***^ | 0.11 ^***^ | 0.1 ^***^ | 0.06 ^***^ | 0.23 ^***^ | 0.17 ^***^ | 0.14 ^***^ | 0.14 ^***^ | 0.12 ^***^ |  |  |  |  |  |  |  |  |  |
| 13 | W3_Pemo | 0.04 | 0.33 | 0.17 ^***^ | 0.25 ^***^ | 0.15 ^***^ | 0.14 ^***^ | 0.12 ^***^ | 0.1 ^***^ | 0.18 ^***^ | 0.26 ^***^ | 0.19 ^***^ | 0.17 ^***^ | 0.16 ^***^ | 0.43 ^***^ |  |  |  |  |  |  |  |  |
| 14 | W4_Pemo | 0.03 | 0.33 | 0.16 ^***^ | 0.21 ^***^ | 0.3 ^***^ | 0.2 ^***^ | 0.17 ^***^ | 0.15 ^***^ | 0.19 ^***^ | 0.23 ^***^ | 0.29 ^***^ | 0.23 ^***^ | 0.21 ^***^ | 0.34 ^***^ | 0.5 ^***^ |  |  |  |  |  |  |  |
| 15 | W5_Pemo | 0.03 | 0.36 | 0.1 ^***^ | 0.18 ^***^ | 0.21 ^***^ | 0.4 ^***^ | 0.29 ^***^ | 0.22 ^***^ | 0.19 ^***^ | 0.23 ^***^ | 0.26 ^***^ | 0.36 ^***^ | 0.29 ^***^ | 0.26 ^***^ | 0.38 ^***^ | 0.48 ^***^ |  |  |  |  |  |  |
| 16 | W6_Pemo | 0.03 | 0.36 | 0.1 ^***^ | 0.14 ^***^ | 0.18 ^***^ | 0.25 ^***^ | 0.39 ^***^ | 0.27 ^***^ | 0.18 ^***^ | 0.21 ^***^ | 0.22 ^***^ | 0.27 ^***^ | 0.35 ^***^ | 0.23 ^***^ | 0.33 ^***^ | 0.4 ^***^ | 0.54 ^***^ |  |  |  |  |  |
| 17 | W4_Yemo | 0 | 0.29 | -0.01 | 0.01 | 0.07 ^***^ | 0.05 ^***^ | 0.01 | 0.01 | 0.03 ^**^ | 0.04 ^***^ | 0.06 ^***^ | 0.05 ^***^ | 0.06 ^***^ | 0 | 0.03 ^*^ | 0.08 ^***^ | 0.06 ^***^ | 0.05 ^***^ |  |  |  |  |
| 18 | W5_Yemo | 1.99 | 0.72 | 0.02 | 0.06 ^***^ | 0.11 ^***^ | 0.22 ^***^ | 0.16 ^***^ | 0.12 ^***^ | 0.09 ^***^ | 0.1 ^***^ | 0.13 ^***^ | 0.18 ^***^ | 0.15 ^***^ | 0.03 ^**^ | 0.07 ^***^ | 0.09 ^***^ | 0.23 ^***^ | 0.17 ^***^ | 0.13 ^***^ |  |  |  |
| 19 | W6_Yemo | 1.43 | 0.45 | 0.01 | 0.03 ^*^ | 0.05 ^***^ | 0.11 ^***^ | 0.17 ^***^ | 0.15 ^***^ | 0.04 ^***^ | 0.03 ^**^ | 0.04 ^***^ | 0.07 ^***^ | 0.1 ^***^ | 0.01 | 0.05 ^***^ | 0.07 ^***^ | 0.13 ^***^ | 0.27 ^***^ | 0.07 ^***^ | 0.25 ^***^ |  |  |
| 20 | W5_SE | 3.39 | 0.43 | -0.03 ^*^ | -0.04 ^***^ | -0.08 ^***^ | -0.14 ^***^ | -0.13 ^***^ | -0.11 ^***^ | -0.07 ^***^ | -0.1 ^***^ | -0.11 ^***^ | -0.15 ^***^ | -0.13 ^***^ | -0.03 ^*^ | -0.06 ^***^ | -0.09 ^***^ | -0.17 ^***^ | -0.14 ^***^ | -0.07 ^***^ | -0.36 ^***^ | -0.2 ^***^ |  |
| 21 | W6_SE | 3.12 | 0.58 | -0.04 ^**^ | -0.01 | -0.05 ^***^ | -0.08 ^***^ | -0.11 ^***^ | -0.1 ^***^ | -0.03 ^*^ | -0.03 ^**^ | -0.03 ^*^ | -0.05 ^***^ | -0.08 ^***^ | -0.01 | -0.06 ^***^ | -0.06 ^***^ | -0.12 ^***^ | -0.23 ^***^ | -0.05 ^***^ | -0.18 ^***^ | -0.6 ^***^ | 0.3 ^***^ |
| *Note.* *W* Wave, *Vic* Victimization, *Hyp* Hyperactive/inattention Problems, *Pemo* Parent-reported Emotional Problems, *Yemo* Youth-reported Emotional Problems*, SE* Self-Esteem.  **p* < .05, ***p* < .01, ****p* < .001. | | | | | | | | | | | | | | | | | | | | | | | |

| Table S8 | | | | | | | | | | | | | | | | | | | | | | | | | | |
| --- | --- | --- | --- | --- | --- | --- | --- | --- | --- | --- | --- | --- | --- | --- | --- | --- | --- | --- | --- | --- | --- | --- | --- | --- | --- | --- |
| *Correlations Between Victimization and Associated Relational Factors* | | | | | | | | | | | | | | | | | | | | | | | | | | |
| Variable | | Mean | *SD* | 1 | 2 | 3 | 4 | 5 | 6 | 7 | 8 | 9 | 10 | 11 | 12 | 13 | 14 | 15 | 16 | 17 | 18 | 19 | 20 | 21 | 22 | 23 |
| 1 | W2_Vic | 1.09 | 0.32 |  |  |  |  |  |  |  |  |  |  |  |  |  |  |  |  |  |  |  |  |  |  |  |
| 2 | W3_Vic | 1.15 | 0.41 | 0.19 ^***^ |  |  |  |  |  |  |  |  |  |  |  |  |  |  |  |  |  |  |  |  |  |  |
| 3 | W4_Vic | 1.22 | 0.48 | 0.13 ^***^ | 0.28 ^***^ |  |  |  |  |  |  |  |  |  |  |  |  |  |  |  |  |  |  |  |  |  |
| 4 | W5_Vic | 1.28 | 0.54 | 0.1 ^***^ | 0.23 ^***^ | 0.31 ^***^ |  |  |  |  |  |  |  |  |  |  |  |  |  |  |  |  |  |  |  |  |
| 5 | W6_Vic | 1.28 | 0.56 | 0.08 ^***^ | 0.17 ^***^ | 0.23 ^***^ | 0.4 ^***^ |  |  |  |  |  |  |  |  |  |  |  |  |  |  |  |  |  |  |  |
| 6 | W7_Vic | 1.2 | 0.49 | 0.08 ^***^ | 0.13 ^***^ | 0.2 ^***^ | 0.31 ^***^ | 0.41 ^***^ |  |  |  |  |  |  |  |  |  |  |  |  |  |  |  |  |  |  |
| 7 | W2_PP | 0.03 | 0.45 | 0.21 ^***^ | 0.14 ^***^ | 0.13 ^***^ | 0.12 ^***^ | 0.1 ^***^ | 0.08 ^***^ |  |  |  |  |  |  |  |  |  |  |  |  |  |  |  |  |  |
| 8 | W3_PP | 0.04 | 0.43 | 0.14 ^***^ | 0.23 ^***^ | 0.19 ^***^ | 0.14 ^***^ | 0.14 ^***^ | 0.12 ^***^ | 0.43 ^***^ |  |  |  |  |  |  |  |  |  |  |  |  |  |  |  |  |
| 9 | W4_PP | 0.04 | 0.42 | 0.14 ^***^ | 0.2 ^***^ | 0.31 ^***^ | 0.23 ^***^ | 0.19 ^***^ | 0.16 ^***^ | 0.36 ^***^ | 0.52 ^***^ |  |  |  |  |  |  |  |  |  |  |  |  |  |  |  |
| 10 | W5_PP | 0.04 | 0.45 | 0.13 ^***^ | 0.17 ^***^ | 0.23 ^***^ | 0.41 ^***^ | 0.28 ^***^ | 0.23 ^***^ | 0.3 ^***^ | 0.39 ^***^ | 0.49 ^***^ |  |  |  |  |  |  |  |  |  |  |  |  |  |  |
| 11 | W6_PP | 0.02 | 0.45 | 0.11 ^***^ | 0.17 ^***^ | 0.22 ^***^ | 0.31 ^***^ | 0.38 ^***^ | 0.27 ^***^ | 0.27 ^***^ | 0.34 ^***^ | 0.42 ^***^ | 0.53 ^***^ |  |  |  |  |  |  |  |  |  |  |  |  |  |
| 12 | W4_Ex | 1.65 | 0.61 | 0.02 | 0.04 ^***^ | 0.1 ^***^ | 0.1 ^***^ | 0.05 ^***^ | 0.04 ^**^ | 0.01 | 0.03 ^*^ | 0.07 ^***^ | 0.07 ^***^ | 0.08 ^***^ |  |  |  |  |  |  |  |  |  |  |  |  |
| 13 | W4_AF | 2.53 | 0.67 | -0.02 ^*^ | -0.06 ^***^ | -0.1 ^***^ | -0.09 ^***^ | -0.06 ^***^ | -0.04 ^**^ | -0.05 ^***^ | -0.09 ^***^ | -0.14 ^***^ | -0.13 ^***^ | -0.13 ^***^ | -0.12 ^***^ |  |  |  |  |  |  |  |  |  |  |  |
| 14 | W5_AF | 3.86 | 1.21 | -0.04 ^**^ | -0.02 | -0.05 ^***^ | -0.05 ^***^ | -0.04 ^***^ | -0.05 ^***^ | -0.08 ^***^ | -0.09 ^***^ | -0.12 ^***^ | -0.18 ^***^ | -0.14 ^***^ | -0.03 ^*^ | 0.09 ^***^ |  |  |  |  |  |  |  |  |  |  |
| 15 | W6_AF | 3.8 | 1.24 | -0.02 | -0.03 ^**^ | -0.06 ^***^ | -0.07 ^***^ | -0.1 ^***^ | -0.1 ^***^ | -0.09 ^***^ | -0.1 ^***^ | -0.13 ^***^ | -0.19 ^***^ | -0.25 ^***^ | -0.02 | 0.09 ^***^ | 0.28 ^***^ |  |  |  |  |  |  |  |  |  |
| 16 | W4_SoD | 0.86 | 0.34 | 0 | -0.01 | 0 | -0.02 | 0.01 | 0 | -0.02 | -0.01 | -0.02 | -0.03 ^**^ | -0.04 ^**^ | -0.01 | 0.05 ^***^ | 0 | 0.01 |  |  |  |  |  |  |  |  |
| 17 | W5_SoD | 0.89 | 0.31 | -0.01 | -0.02 | -0.03 ^**^ | -0.05 ^***^ | -0.03 ^**^ | -0.01 | -0.04 ^***^ | -0.04 ^***^ | -0.07 ^***^ | -0.08 ^***^ | -0.07 ^***^ | -0.03 ^**^ | 0.06 ^***^ | 0.05 ^***^ | 0.05 ^***^ | 0.05 ^***^ |  |  |  |  |  |  |  |
| 18 | W6_SoD | 0.75 | 0.43 | -0.01 | -0.04 ^***^ | -0.03 ^**^ | -0.04 ^***^ | -0.02 | -0.02 | -0.02 ^*^ | -0.03 ^*^ | -0.03 ^**^ | -0.05 ^***^ | -0.07 ^***^ | -0.03 ^**^ | 0.04 ^***^ | 0.04 ^***^ | 0.09 ^***^ | 0.03 ^*^ | 0.16 ^***^ |  |  |  |  |  |  |
| 19 | W6_SS | 2.83 | 0.3 | -0.03 ^*^ | -0.05 ^***^ | -0.05 ^***^ | -0.08 ^***^ | -0.08 ^***^ | -0.06 ^***^ | -0.06 ^***^ | -0.07 ^***^ | -0.09 ^***^ | -0.11 ^***^ | -0.16 ^***^ | -0.06 ^***^ | 0.07 ^***^ | 0.06 ^***^ | 0.17 ^***^ | 0.02 | 0.12 ^***^ | 0.4 ^***^ |  |  |  |  |  |
| 20 | W3_Pcl | 3.66 | 0.55 | -0.08 ^***^ | -0.05 ^***^ | -0.04 ^***^ | -0.03 ^*^ | -0.03 ^**^ | -0.01 | -0.11 ^***^ | -0.16 ^***^ | -0.12 ^***^ | -0.11 ^***^ | -0.11 ^***^ | -0.03 ^*^ | 0.04 ^***^ | 0.03 ^**^ | 0.04 ^***^ | 0 | 0.04 ^***^ | 0.03 ^*^ | 0.06 ^***^ |  |  |  |  |
| 21 | W4_Pcl | 3.61 | 0.58 | -0.07 ^***^ | -0.03 ^**^ | -0.05 ^***^ | -0.03 ^**^ | -0.02 | -0.02 | -0.09 ^***^ | -0.1 ^***^ | -0.13 ^***^ | -0.09 ^***^ | -0.08 ^***^ | -0.04 ^***^ | 0.04 ^***^ | 0.03 ^**^ | 0.04 ^**^ | 0.01 | 0.05 ^***^ | 0.05 ^***^ | 0.06 ^***^ | 0.48 ^***^ |  |  |  |
| 22 | W5_Pcl | 3.52 | 0.62 | -0.03 ^*^ | -0.01 | -0.04 ^***^ | -0.03 ^**^ | -0.02 ^*^ | -0.01 | -0.09 ^***^ | -0.1 ^***^ | -0.11 ^***^ | -0.12 ^***^ | -0.1 ^***^ | -0.03 ^*^ | 0.04 ^***^ | 0.02 ^*^ | 0.03 ^**^ | 0.02 | 0.04 ^***^ | 0.05 ^***^ | 0.09 ^***^ | 0.38 ^***^ | 0.46 ^***^ |  |  |
| 23 | W6_Pcl | 3.33 | 0.71 | -0.05 ^***^ | -0.01 | -0.03 ^**^ | -0.01 | -0.04 ^***^ | -0.01 | -0.08 ^***^ | -0.08 ^***^ | -0.07 ^***^ | -0.08 ^***^ | -0.14 ^***^ | -0.04 ^***^ | 0.04 ^***^ | 0.01 | 0 | 0.02 | 0.04 ^***^ | 0.1 ^***^ | 0.15 ^***^ | 0.3 ^***^ | 0.37 ^***^ | 0.48 ^***^ |  |
| 24 | W6_Ycl | 2.98 | 0.83 | -0.05 ^***^ | -0.07 ^***^ | -0.07 ^***^ | -0.07 ^***^ | -0.07 ^***^ | -0.09 ^***^ | -0.07 ^***^ | -0.09 ^***^ | -0.1 ^***^ | -0.12 ^***^ | -0.15 ^***^ | -0.06 ^***^ | 0.04 ^***^ | 0.02 | 0.02 ^*^ | -0.01 | 0.07 ^***^ | 0.21 ^***^ | 0.32 ^***^ | 0.06 ^***^ | 0.07 ^***^ | 0.11 ^***^ | 0.2 ^***^ |
| *Note.* *W* Wave, *Vic* Victimization, *PP* Peer Problems, *Ex* Peer Exclusion, *AF* Availability of Friendships, *SoD* Social Disclosure, *SS* Social Support, *Pcl* Parent-reported Parent-child Closeness, *Ycl* Youth-reported Parent-child Closeness.  **p* < .05, ***p* < .01, ****p* < .001. | | | | | | | | | | | | | | | | | | | | | | | | | | |

| Table S9 | | | | | | | | | | | | | | | | |
| --- | --- | --- | --- | --- | --- | --- | --- | --- | --- | --- | --- | --- | --- | --- | --- | --- |
| *Correlations Between Victimization and Health and Well-Being Outcomes* | | | | | | | | | | | | | | | | |
| Variable | | Mean | *SD* | 1 | 2 | 3 | 4 | 5 | 6 | 7 | 8 | 9 | 10 | 11 | 12 | 13 |
| 1 | W2_Vic | 1.09 | 0.32 |  |  |  |  |  |  |  |  |  |  |  |  |  |
| 2 | W3_Vic | 1.15 | 0.41 | 0.19 ^***^ |  |  |  |  |  |  |  |  |  |  |  |  |
| 3 | W4_Vic | 1.22 | 0.48 | 0.13 ^***^ | 0.28 ^***^ |  |  |  |  |  |  |  |  |  |  |  |
| 4 | W5_Vic | 1.28 | 0.54 | 0.1 ^***^ | 0.23 ^***^ | 0.31 ^***^ |  |  |  |  |  |  |  |  |  |  |
| 5 | W6_Vic | 1.28 | 0.56 | 0.08 ^***^ | 0.17 ^***^ | 0.23 ^***^ | 0.4 ^***^ |  |  |  |  |  |  |  |  |  |
| 6 | W7_Vic | 1.2 | 0.49 | 0.08 ^***^ | 0.13 ^***^ | 0.2 ^***^ | 0.31 ^***^ | 0.41 ^***^ |  |  |  |  |  |  |  |  |
| 7 | W7_SH | 0.45 | 0.98 | 0.01 | 0.02 ^*^ | 0.06 ^***^ | 0.12 ^***^ | 0.13 ^***^ | 0.15 ^***^ |  |  |  |  |  |  |  |
| 8 | W7_Emo | 2.21 | 0.82 | 0.02 | 0.03 ^**^ | 0.06 ^***^ | 0.11 ^***^ | 0.12 ^***^ | 0.15 ^***^ | 0.52 ^***^ |  |  |  |  |  |  |
| 9 | W7_GH | 3.89 | 0.93 | -0.05 ^***^ | -0.09 ^***^ | -0.1 ^***^ | -0.14 ^***^ | -0.13 ^***^ | -0.15 ^***^ | -0.2 ^***^ | -0.29 ^***^ |  |  |  |  |  |
| 10 | W7_SBH | 0.03 | 0.17 | 0.01 | 0.09 ^***^ | 0.1 ^***^ | 0.18 ^***^ | 0.19 ^***^ | 0.21 ^***^ | 0.14 ^***^ | 0.15 ^***^ | -0.15 ^***^ |  |  |  |  |
| 11 | W7_Smo | 2.09 | 1.6 | -0.01 | 0.01 | 0.03 ^*^ | 0.05 ^***^ | 0.04 ^***^ | 0.02 | 0.22 ^***^ | 0.18 ^***^ | -0.11 ^***^ | 0.02 |  |  |  |
| 12 | W7_Vap | 1.92 | 1.24 | -0.01 | 0.02 | 0.03 ^*^ | 0.03 ^*^ | 0.04 ^***^ | 0.02 | 0.16 ^***^ | 0.12 ^***^ | -0.07 ^***^ | 0.02 | 0.6 ^***^ |  |  |
| 13 | W7_Alco | 1.88 | 1.44 | -0.07 ^***^ | -0.07 ^***^ | -0.08 ^***^ | -0.06 ^***^ | -0.07 ^***^ | -0.07 ^***^ | 0.11 ^***^ | 0.09 ^***^ | 0.06 ^***^ | -0.05 ^***^ | 0.42 ^***^ | 0.32 ^***^ |  |
| 14 | W7_Cnb | 0.53 | 1.09 | -0.03 ^*^ | -0.04 ^***^ | -0.03 ^**^ | -0.04 ^***^ | -0.04 ^***^ | -0.04 ^***^ | 0.16 ^***^ | 0.13 ^***^ | -0.02 ^*^ | -0.01 | 0.49 ^***^ | 0.39 ^***^ | 0.34 ^***^ |
| *Note.* *W* Wave, *Vic* Victimization, *SH* Self-harm, *Emo* Emotional Problems*, GH* General Health*, SBH* Social and Behavioural Concerns*, Smo* Smoking*, Vap* Vaping*, Alco* Alcohol Use*, Cnb* Cannabis Use*.*  **p* < .05, ***p* < .01, ****p* < .001. | | | | | | | | | | | | | | | | |

# **Appendix F: Longitudinal Measurement Invariance**

Given that the Chi-square test is sensitive to sample size and likely to be significant for large samples, the ΔCFIs and ΔRMSEAs were used as indicators for longitudinal measurement invariance. A decrease in CFI less than 0.01 (Cheung & Rensvold, 2002) and an increase in RMSEA less than 0.015 (Chen, 2007) are considered to indicate acceptable invariance.

| Table S10 | | | | | |
| --- | --- | --- | --- | --- | --- |
| *Longitudinal Measurement Invariance* | | | | | |
| Variable | Model | CFI | ΔCFI | RMSEA | ΔRMSEA |
|  |  |  |  |  |  |
| Emotional problems (Parent-reported) | Configual | 0.981 | - | 0.025 | - |
|  | Metric | 0.978 | **-0.003** | 0.027 | **0.002** |
|  | Scalar | 0.893 | -0.085 | 0.054 | 0.027 |
| Hyperactive/inattention problems  (Parent-reported) | Configual | 0.943 | - | 0.067 | - |
|  | Metric | 0.949 | 0.006 | 0.061 | -0.006 |
|  | Scalar | 0.939 | **-0.010** | 0.061 | **0** |
| Peer problems  (Parent-reported) | Configual | 0.961 | - | 0.039 | - |
|  | Metric | 0.96 | **-0.001** | 0.036 | -0.003 |
|  | Scalar | 0.925 | -0.035 | 0.046 | **0.010** |
| Self-esteem  (Youth-reported) | Configual | 0.969 | - | 0.066 | - |
|  | Metric | 0.968 | **-0.001** | 0.061 | -0.005 |
|  | Scalar | 0.949 | -0.019 | 0.074 | **0.013** |
| *Note.* Bold indicates the model comparison results showed acceptable invariance | | | | | |

**References**

Chen, F. F. (2007). Sensitivity of goodness of fit indexes to lack of measurement invariance. *Structural Equation Modeling: A Multidisciplinary Journal*, *14*, 464-504. https://doi.org/10.1080/10705510701301834.

Cheung, G. W., & Rensvold, R. B. (2002). Evaluating goodness-of-fit indexes for testing measurement invariance. *Structural Equation Modeling: A Multidisciplinary Journal*, *9*, 233-255. [https://doi.org/10.1207/S15328007SEM0902_5](http://dx.doi.org/10.1207/S15328007SEM0902_5).

# **Appendix G: Model Fit Indices for Latent Class Growth Models**

| Table S11 | | | | | | | | |
| --- | --- | --- | --- | --- | --- | --- | --- | --- |
| *Model Fit Indices for Quadric Latent Class Growth Models* | | | | | | | | |
|  | AIC | BIC | aBIC | Entropy | *p*(LMR-LRT) | *p*(aLRT) | *p*(BLRT) | Percentage of each class |
| 1 | 66381.87 | 66446.84 | 66418.24 | - | - | - | - | - |
| 2 | 50517.78 | 50611.62 | 50570.31 | 0.960 | 0.0043 | 0.0048 | 0 | 91.8%, 8.2% |
| 3 | 42164.46 | 42287.17 | 42233.15 | 0.948 | 0 | 0 | 0 | 79.1%, 13.5%, 7.5% |
| **4** | **40111.59** | **40263.17** | **40196.44** | **0.923** | **0** | **0** | **0** | **73.6%, 12.8%, 7.2%, 6.3%** |
| 5 | 38190.27 | 38370.73 | 38291.28 | 0.913 | 0.0014 | 0.0016 | 0 | 71.5%, 9.1%, 7.9%, 7.2%, 4.2% |
| *Note.* Bold indicates the final class solution. *AIC* Akaike Information Criterion, *BIC* Bayesian Information Criteria, *aBIC* aBIC Sample Size Adjusted Bayesian Information Criteria, *LRT* Vuong-Lo-Mendell-Rubin Likelihood Ratio Test, *aLRT* Adjusted Lo-Mendell-Rubin Likelihood Test, *BLRT* Bootstrap Likelihood Ratio Tests. | | | | | | | | |

# **Appendix H: Intercept, Slope, and Quadratic Factors of the 4-Class Trajectory**

| Table S12 | | | |
| --- | --- | --- | --- |
| *Intercept, Slope, and Quadratic Factors of the 4-Class Trajectory* | | | |
| Trajectory | Factors | Means | 95% CI |
| Low | I | 1.12 | 1.11, 1.13 |
|  | S | 0.01 | 0.01, 0.01 |
|  | Q | 0 | 0, 0 |
| Early peak | I | 1.51 | 1.47, 1.55 |
|  | S | -0.09 | -0.09, -0.08 |
|  | Q | 0.01 | 0.01, 0.01 |
| Late childhood peak | I | 2.07 | 2.01, 2.12 |
|  | S | 0.11 | 0.10, 0.11 |
|  | Q | -0.02 | -0.02, -0.02 |
| Adolescence onset | I | 1.37 | 1.34, 1.41 |
|  | S | 0.08 | 0.08, 0.08 |
|  | Q | 0 | 0, 0 |
| *Note.* *I* Intercept, *S* Slope, *Q* Quadratic Factors. | | | |

# **Appendix I: Indirect Effect of Associated Factors**

| Table S13 | | | | | | | |
| --- | --- | --- | --- | --- | --- | --- | --- |
| *Indirect Effects of Self-Esteem, Peer Exclusion, Social Disclosure, and Social Support Between SGD Status and Victimization Class Membership* | | | | | | | |
| Mediator | Wave | Class membership | | SA_D | | SI_D | |
|  |  | Reference | Outcome | b | 95% CI | b | 95% CI |
| Self-esteem | 5 | Low | EP | .02* | 0, .03 | .02* | 0, .03 |
|  |  |  | LCP | .03* | .01, .05 | .03* | .01, .05 |
|  |  |  | AO | .04* | .02, .06 | .04* | .02, .06 |
|  |  | AO | EP | - | - | -.02* | -.04, 0 |
|  | 6 | Low | EP | .05* | .02, .08 | .07* | .03, .11 |
|  |  |  | LCP | .05* | .02, .08 | .06* | .02, .1 |
|  |  |  | AO | .07* | .04, .10 | .09* | .06, .12 |
|  |  | AO | EP | - | - | -.02 | -.06, .02 |
| Peer exclusion | 4 | Low | EP | .01 | 0, .02 | .01 | 0, .02 |
|  |  |  | LCP | .01 | 0, .03 | .02 | 0, .03 |
|  |  |  | AO | .01 | 0, .02 | .01 | 0, .02 |
|  |  | AO | EP | - | - | 0 | -.01, .01 |
| Social disclosure | 4 | Low | EP | 0 | 0, 0 | 0 | 0, 0 |
|  |  |  | LCP | 0 | 0, 0 | 0 | 0, 0 |
|  |  |  | AO | 0 | 0, 0 | 0 | 0, 0 |
|  |  | AO | EP | - | - | 0 | 0, 0 |
|  | 5 | Low | EP | 0 | 0, .01 | 0 | -.01, .01 |
|  |  |  | LCP | 0 | 0, .01 | 0 | 0, .01 |
|  |  |  | AO | 0 | 0, .01 | .01 | 0, .01 |
|  |  | AO | EP | - | - | 0 | -.01, 0 |
|  | 6 | Low | EP | .01 | 0, .03 | .02 | 0, .04 |
|  |  |  | LCP | .01 | 0, .03 | .02 | -.01, .04 |
|  |  |  | AO | .02 | 0, .03 | .02 | 0, .04 |
|  |  | AO | EP | - | - | 0 | -.03, .02 |
| Social support | 6 | Low | EP | .03* | 0, .06 | .03* | .01, .06 |
|  |  |  | LCP | .05* | .02, .08 | .05* | .02, .08 |
|  |  |  | AO | .06* | .03, .08 | .05* | .03, .08 |
|  |  | AO | EP | - | - | -.02 | -.05, .01 |
| Mediator | Wave | Class membership | | SA_M | | SI_M | |
|  |  | Reference | Outcome | b | 95% CI | b | 95% CI |
| Self-esteem | 5 | Low | AO | .03* | .01, .05 | .02* | .01, .04 |
|  | 6 |  | AO | .08* | .05, .10 | .05* | .03, .08 |
| Peer exclusion | 4 | Low | AO | .01* | 0, .02 | .01* | 0, .02 |
| Social disclosure | 4 | Low | AO | 0 | 0, 0 | 0 | 0, 0 |
|  | 5 |  | AO | 0 | 0, .01 | 0 | 0, .01 |
|  | 6 |  | AO | .01 | 0, .02 | .01 | 0, .01 |
| Social support | 6 | Low | AO | .03* | .02, .05 | .02* | .01, .04 |
| *Note*. *SA_D* Sexual Attraction Diverse, *SA_M* Mostly Other-Sex Attracted, *SI_D* Sexual Identity Diverse, *SI_M* Mainly Heterosexual, *Low* Low Victimization Class, *EP* Early Peak Victimization Class, *LCP* Late Childhood Peak Victimization Class, *AO* Adolescence Onset Victimization Class.  *Significant results after correcting for α level for multiple testing. | | | | | | | |

# **Appendix J: Main Effects of Associated Factors from SGD Status to Victimization Trajectories**

| Table S14 | | | | | | | | | | | |
| --- | --- | --- | --- | --- | --- | --- | --- | --- | --- | --- | --- |
| *Main Effects of Sexual Attraction and Associated Mental Health Factors, Using Low Victimization Class as the Reference Class* | | | | | | | | | | | |
| Mediator | Wave | SA_D → Mediator | | SA_M → Mediator | | Mediator → EP | | Mediator → LCP | | Mediator → AO | |
|  |  | *β* | 95% CI | *β* | 95% CI | *β* | 95% CI | *β* | 95% CI | *β* | 95% CI |
| Emotional problems (Parent-reported) | 2 | .01 | -.02, .03 | -.03* | -.05, -.01 | .72* | .64, .80 | .33* | .24, .42 | .12* | .06, .19 |
|  | 3 | .03* | .01, .05 | -.01 | -.03, .01 | .54* | .47, .62 | .44* | .36, .53 | .25* | .19, .32 |
|  | 4 | .05* | .02, .07 | .01 | -.01, .03 | .56* | .48, .64 | .62* | .54, .71 | .40* | .34, .46 |
|  | 5 | .07* | .05, .10 | .02 | 0, .04 | .47* | .39, .55 | .72* | .64, .81 | .66* | .60, .73 |
|  | 6 | .10* | .08, .12 | .03* | .01, .05 | .45* | .36, .53 | .68* | .59, .76 | .77* | .71, .84 |
| Emotional problems  (Youth-reported) | 4 | .03* | .01, .05 | .05* | .03, .08 | .02 | -.07, .10 | .18* | .08, .28 | .04 | -.03, .11 |
|  | 5 | .07* | .05, .10 | .07* | .05, .09 | .12* | .04, .20 | .37* | .29, .45 | .37* | .31, .43 |
|  | 6 | .14* | .12, .16 | .16* | .14, .18 | .11* | .03, .20 | .24* | .15, .33 | .38* | .32, .44 |
| Hyperactive/inattention problems | 2 | .03* | .01, .05 | -.02 | -.04, 0 | .31* | .23, .39 | .33* | .23, .42 | .26* | .20, .33 |
|  | 3 | .05* | .03, .07 | .01 | -.01, .02 | .34* | .26, .42 | .44* | .35, .53 | .35* | .29, .41 |
|  | 4 | .07* | .05, .09 | .01 | -.01, .03 | .29* | .21, .38 | .48* | .39, .57 | .44* | .37, .50 |
|  | 5 | .06* | .04, .08 | .02 | 0, .04 | .32* | .24, .41 | .55* | .46, .63 | .53* | .47, .60 |
|  | 6 | .03* | .01, .06 | .01 | -.02, .03 | .31* | .23, .39 | .45* | .36, .53 | .55* | .48, .61 |
| Self-esteem | 5 | -.04* | -.06, -.02 | -.04* | -.06, -.02 | -.14* | -.22, -.05 | -.20* | -.28, -.11 | -.28* | -.34, -.22 |
|  | 6 | -.09* | -.11, -.07 | -.11* | -.13, -.09 | -.18* | -.27, -.09 | -.17* | -.26, -.07 | -.24* | -.30, -.17 |
| *Note*. *SA_D* Sexual Attraction Diverse, *SA_M* Mostly other-sex attracted, *EP* Early Peak Victimization Class, *LCP* Late Childhood Peak Victimization Class, *AO* Adolescence Onset Victimization Class.  *Significant results after correcting for α level for multiple testing. | | | | | | | | | | | |

| Table S15 | | | | | | | | | | | |
| --- | --- | --- | --- | --- | --- | --- | --- | --- | --- | --- | --- |
| *Main Effects of Sexual Identity and Associated Mental Health Factors, Using Low Victimization Class as the Reference Class* | | | | | | | | | | | |
| Mediator | Wave | SI_D→ Mediator | | SI_M → Mediator | | Mediator → EP | | Mediator → LCP | | Mediator → AO | |
|  |  | *β* | 95% CI | *β* | 95% CI | *β* | 95% CI | *β* | 95% CI | *β* | 95% CI |
| Emotional problems (Parent-reported) | 2 | .01 | -.01, .03 | -.03* | -.05, -.01 | .71* | .63, .79 | .33* | .24, .43 | .12* | .05, .19 |
|  | 3 | .01 | -.01, .03 | -.01 | -.03, .01 | .55* | .47, .62 | .44* | .36, .53 | .26* | .19, .32 |
|  | 4 | .04* | .01, .06 | .01 | -.01, .03 | .56* | .48, .64 | .63* | .54, .71 | .40* | .34, .47 |
|  | 5 | .07* | .05, .10 | .02 | 0, .04 | .47* | .39, .55 | .72* | .64, .81 | .66* | .60, .73 |
|  | 6 | .10* | .07, .12 | .02 | 0, .04 | .45* | .36, .54 | .67* | .58, .76 | .77* | .71, .84 |
| Emotional problems (Youth-reported) | 4 | .05* | .03, .07 | .04* | .02, .06 | .01 | -.07, .10 | .18* | .08, .28 | .04 | -.04, .11 |
|  | 5 | .08* | .06, .10 | .06* | .04, .08 | .13* | .05, .21 | .37* | .29, .45 | .37* | .31, .43 |
|  | 6 | .18* | .16, .20 | .10* | .08, .12 | .11* | .03, .19 | .23* | .14, .32 | .36* | .30, .42 |
| Hyperactive/inattention problems | 2 | .02 | -.01, .04 | -.02 | -.04, 0 | .31* | .23, .40 | .33* | .24, .43 | .27* | .20, .34 |
|  | 3 | .03* | .01, .05 | -.01 | -.03, .01 | .34* | .26, .42 | .44* | .35, .53 | .35* | .29, .42 |
|  | 4 | .06* | .04, .08 | -.01 | -.03, .01 | .29* | .21, .38 | .49* | .40, .57 | .44* | .38, .50 |
|  | 5 | .06* | .04, .08 | -.01 | -.03, .01 | .33* | .24, .41 | .54* | .46, .63 | .53* | .47, .59 |
|  | 6 | .04* | .02, .06 | -.01 | -.03, .01 | .31* | .23, .39 | .44* | .36, .53 | .55* | .48, .61 |
| Self-esteem | 5 | -.04* | -.06, -.02 | -.03* | -.05, -.01 | -.14* | -.22, -.06 | -.20* | -.28, -.11 | -.28* | -.34, -.22 |
|  | 6 | -.12* | -.14, -.10 | -.07* | -.09, -.05 | -.18* | -.27, -.10 | -.16* | -.25, -.07 | -.23* | -.30, -.16 |
| *Note*. *SI_D* Sexual Identity Diverse, *SI_M* Mainly Heterosexual, *EP* Early Peak Victimization Class, *LCP* Late Childhood Peak Victimization Class, *AO* Adolescence Onset Victimization Class.  *Significant results after correcting for α level for multiple testing. | | | | | | | | | | | |

| Table S16 | | | | | | | | | | | |
| --- | --- | --- | --- | --- | --- | --- | --- | --- | --- | --- | --- |
| *Main Effects of Sexual Attraction and Associated Relational Factors, Using Low Victimization Class as the Reference Class* | | | | | | | | | | | |
| Mediator | Wave | SA_D → Mediator | | SA_M → Mediator | | Mediator → EP | | Mediator → LCP | | Mediator → AO | |
|  |  | *β* | 95% CI | *β* | 95% CI | *β* | 95% CI | *β* | 95% CI | *β* | 95% CI |
| Peer problems | 2 | .05* | .02, .07 | 0 | -.02, .02 | .62* | .54, .70 | .30* | .20, .39 | .17* | .11, .24 |
|  | 3 | .08* | .05, .10 | .02* | 0, .04 | .46* | .38, .54 | .42* | .33, .5 | .28* | .22, .34 |
|  | 4 | .10* | .08, .13 | .03* | .01, .05 | .48* | .40, .56 | .65* | .57, .74 | .46* | .40, .53 |
|  | 5 | .14* | .12, .16 | .05* | .03, .07 | .49* | .41, .57 | .73* | .65, .82 | .70* | .64, .77 |
|  | 6 | .16* | .14, .18 | .07* | .05, .09 | .48* | .40, .57 | .68* | .59, .77 | .82* | .75, .89 |
| Availability of friendships | 4 | -.05* | -.07, -.03 | -.06* | -.09, -.04 | -.09 | -.17, -.01 | -.20* | -.29, -.11 | -.10* | -.17, -.04 |
|  | 5 | -.04* | -.07, -.02 | -.06* | -.08, -.04 | -.12* | -.20, -.04 | -.14* | -.22, -.05 | -.19* | -.25, -.13 |
|  | 6 | -.13* | -.15, -.11 | -.04* | -.06, -.02 | -.04 | -.12, .05 | -.04 | -.13, .05 | -.26* | -.32, -.20 |
| Peer exclusion | 4 | .02 | 0, .04 | .04* | .02, .06 | .13* | .04, .21 | .21* | .12, .31 | .12* | .05, .18 |
| Parent-child closeness (Parent-reported) | 3 | -.01 | -.03, .01 | .01 | -.01, .03 | -.19* | -.27, -.12 | .02 | -.08, .12 | -.06 | -.12, .01 |
|  | 4 | -.01 | -.03, .01 | 0 | -.02, .02 | -.15* | -.23, -.07 | -.10 | -.19, 0 | -.05 | -.11, .02 |
|  | 5 | -.01 | -.03, .01 | .01 | -.02, .03 | -.06 | -.14, .03 | -.05 | -.14, .04 | -.07 | -.14, -.01 |
|  | 6 | -.02 | -.04, .01 | -.04* | -.06, -.02 | -.16* | -.24, -.08 | -.05 | -.14, .04 | -.08* | -.15, -.02 |
| Parent-child closeness (Youth-reported) | 6 | -.06* | -.08, -.04 | -.09* | -.11, -.06 | -.16* | -.24, -.07 | -.13* | -.22, -.04 | -.20* | -.26, -.14 |
| Social disclosure | 4 | -.01 | -.03, .01 | -.01 | -.03, .01 | .01 | -.08, .09 | -.01 | -.10, .08 | -.02 | -.08, .05 |
|  | 5 | -.02 | -.04, 0 | -.02* | -.04, 0 | -.02 | -.10, .06 | -.05 | -.13, .03 | -.07 | -.13, 0 |
|  | 6 | -.06* | -.08, -.04 | -.05* | -.07, -.02 | -.06 | -.15, .02 | -.07 | -.16, .02 | -.08* | -.15, -.02 |
| Social support | 6 | -.10* | -.13, -.08 | -.07* | -.09, -.04 | -.09* | -.17, -.02 | -.15* | -.23, -.07 | -.17* | -.22, -.11 |
| *Note*. *SA_D* Sexual Attraction Diverse, *SA_M* Mostly Other-Sex Attracted, *EP* Early Peak Victimization Class, *LCP* Late Childhood Peak Victimization Class, *AO* Adolescence Onset Victimization Class.  *Significant results after correcting for α level for multiple testing. | | | | | | | | | | | |

| Table S17 | | | | | | | | | | | |
| --- | --- | --- | --- | --- | --- | --- | --- | --- | --- | --- | --- |
| *Main Effects of Sexual Identity and Associated Relational Factors, Using Low Victimization Class as the Reference Class* | | | | | | | | | | | |
| Mediator | Wave | SI_D→ Mediator | | SI_M → Mediator | | Mediator → EP | | Mediator → LCP | | Mediator → AO | |
|  |  | *β* | 95% CI | *β* | 95% CI | *β* | 95% CI | *β* | 95% CI | *β* | 95% CI |
| Peer problems | 2 | .04* | .02, .06 | .01 | -.01, .03 | .63* | .55, .71 | .29* | .20, .39 | .17* | .11, .24 |
|  | 3 | .06* | .04, .09 | .02* | 0, .04 | .46* | .39, .54 | .41* | .33, .50 | .28* | .22, .35 |
|  | 4 | .10* | .08, .12 | .02* | 0, .04 | .48* | .40, .56 | .65* | .57, .74 | .46* | .39, .53 |
|  | 5 | .13* | .11, .16 | .04* | .02, .06 | .50* | .42, .58 | .73* | .64, .82 | .70* | .64, .77 |
|  | 6 | .16* | .14, .18 | .07* | .05, .09 | .49* | .40, .57 | .68* | .59, .77 | .82* | .75, .89 |
| Availability of friendships | 4 | -.04* | -.06, -.02 | -.04* | -.07, -.02 | -.09* | -.17, -.01 | -.20* | -.29, -.12 | -.11* | -.17, -.05 |
|  | 5 | -.06* | -.08, -.04 | -.05* | -.07, -.03 | -.12* | -.20, -.04 | -.13* | -.22, -.05 | -.19* | -.25, -.12 |
|  | 6 | -.09* | -.12, -.07 | -.06* | -.08, -.04 | -.05 | -.13, .04 | -.05 | -.14, .05 | -.27* | -.33, -.20 |
| Peer exclusion | 4 | .02* | 0, .04 | .03* | .01, .05 | .13* | .05, .21 | .22* | .13, .31 | .12* | .05, .18 |
| Parent-child closeness (Parent-reported) | 3 | -.02 | -.04, .01 | 0 | -.02, .02 | -.19* | -.26, -.12 | .02 | -.07, .12 | -.06 | -.12, .01 |
|  | 4 | 0 | -.02, .02 | 0 | -.02, .02 | -.16* | -.24, -.08 | -.10 | -.19, -.01 | -.06 | -.12, .01 |
|  | 5 | -.02 | -.04, .01 | .01 | -.01, .03 | -.06 | -.14, .03 | -.05 | -.14, .05 | -.07 | -.13, -.01 |
|  | 6 | -.02 | -.04, 0 | -.03* | -.05, -.01 | -.16* | -.24, -.08 | -.05 | -.14, .04 | -.08 | -.14, -.02 |
| Parent-child closeness (Youth-reported) | 6 | -.09 | -.12, -.07 | -.05* | -.07, -.03 | -.15* | -.24, -.07 | -.13* | -.22, -.04 | -.19* | -.25, -.13 |
| Social disclosure | 4 | -.01 | -.03, .01 | -.01 | -.03, .01 | 0 | -.09, .09 | -.01 | -.10, .08 | -.01 | -.08, .05 |
|  | 5 | -.02 | -.04, 0 | -.01 | -.03, .01 | -.02 | -.10, .07 | -.05 | -.13, .03 | -.06 | -.13, 0 |
|  | 6 | -.08* | -.10, -.06 | -.02 | -.04, 0 | -.07 | -.15, .02 | -.06 | -.15, .03 | -.08* | -.14, -.01 |
| Social support | 6 | -.10* | -.13, -.08 | -.04* | -.07, -.02 | -.09* | -.17, -.02 | -.15* | -.23, -.06 | -.16* | -.22, -.11 |
| *Note*. *SI_D* Sexual Identity Diverse, *SI_M* Mainly Heterosexual, *EP* Early Peak Victimization Class, *LCP* Late Childhood Peak Victimization Class, *AO* Adolescence Onset Victimization Class.  *Significant results after correcting for α level for multiple testing. | | | | | | | | | | | |

| Table S18 | | | | | | | |
| --- | --- | --- | --- | --- | --- | --- | --- |
| *Main Effects of Sexual Identity and Associated Factors, Using Adolescence Onset Victimization Class as the Reference Class* | | | | | | | |
| Mediator | Wave | SI_D → Mediator | | SI_M → Mediator | | Mediator → EP | |
|  |  | *β* | 95% CI | *β* | 95% CI | *β* | 95% CI |
| Emotional problems (Parent-reported) | 2 | .01 | -.01, .03 | -.03* | -.05, -.01 | .59* | .50, .69 |
|  | 3 | .01 | -.01, .03 | -.01 | -.03, .01 | .29* | .20, .38 |
|  | 4 | .04* | .01, .06 | .01 | -.01, .03 | .16* | .07, .25 |
|  | 5 | .07* | .05, .10 | .02 | 0, .04 | -.19* | -.29, -.10 |
|  | 6 | .10* | .07, .12 | .02 | 0, .04 | -.32* | -.42, -.22 |
| Emotional problems (Youth-reported) | 4 | .05* | .03, .07 | .04* | .02, .06 | -.02 | -.13, .08 |
|  | 5 | .08* | .06, .10 | .06* | .04, .08 | -.24* | -.33, -.15 |
|  | 6 | .18* | .16, .2 | .10* | .08, .12 | -.25* | -.35, -.16 |
| Hyperactive/inattention problems | 2 | .02 | -.01, .04 | -.02 | -.04, 0 | .04 | -.06, .14 |
|  | 3 | .03* | .01, .05 | -.01 | -.03, .01 | -.02 | -.11, .08 |
|  | 4 | .06* | .04, .08 | -.01 | -.03, .01 | -.15* | -.25, -.05 |
|  | 5 | .06* | .04, .08 | -.01 | -.03, .01 | -.20* | -.30, -.10 |
|  | 6 | .04* | .02, .06 | -.01 | -.03, .01 | -.24* | -.33, -.14 |
| Self-esteem | 5 | -.04* | -.06, -.02 | -.03* | -.05, -.01 | .14* | .05, .24 |
|  | 6 | -.12* | -.14, -.1 | -.07* | -.09, -.05 | .05 | -.06, .15 |
| Peer problems | 2 | .04* | .02, .06 | .01 | -.01, .03 | .45* | .36, .55 |
|  | 3 | .06* | .04, .09 | .02* | 0, .04 | .18* | .09, .27 |
|  | 4 | .10* | .08, .12 | .02* | 0, .04 | .02* | -.07, .12 |
|  | 5 | .13* | .11, .16 | .04* | .02, .06 | -.20* | -.30, -.11 |
|  | 6 | .16* | .14, .18 | .07* | .05, .09 | -.33* | -.43, -.23 |
| Availability of friendships | 4 | -.04* | -.06, -.02 | -.04* | -.07, -.02 | .02 | -.08, .12 |
|  | 5 | -.06* | -.08, -.04 | -.05* | -.07, -.03 | .07 | -.03, .17 |
|  | 6 | -.09* | -.12, -.07 | -.06* | -.08, -.04 | .22* | .12, .32 |
| Peer exclusion | 4 | .02* | 0, .04 | .03* | .01, .05 | .01 | -.09, .12 |
| Parent-child closeness (Parent-reported) | 3 | -.02 | -.04, .01 | 0 | -.02, .02 | -.13* | -.23, -.04 |
|  | 4 | 0 | -.02, .02 | 0 | -.02, .02 | -.10 | -.20, -.01 |
|  | 5 | -.02 | -.04, .01 | .01 | -.01, .03 | .01 | -.08, .11 |
|  | 6 | -.02 | -.04, 0 | -.03* | -.05, -.01 | -.08 | -.18, .02 |
| Parent-child closeness (Youth-reported) | 6 | -.09* | -.12, -.07 | -.05* | -.07, -.03 | .04 | -.06, .14 |
| Social disclosure | 4 | -.01 | -.03, .01 | -.01 | -.03, .01 | .01 | -.09, .11 |
|  | 5 | -.02 | -.04, 0 | -.01 | -.03, .01 | .05 | -.05, .14 |
|  | 6 | -.08* | -.10, -.06 | -.02 | -.04, 0 | .01 | -.09, .11 |
| Social support | 6 | -.10* | -.13, -.08 | -.04* | -.07, -.02 | .07 | -.02, .16 |
| *Note*. *SI_D* Sexual Identity Diverse, *SI_M* Mainly Heterosexual, *EP* Early Peak Victimization Class.  *Significant results after correcting for α level for multiple testing. | | | | | | | |

# **Appendix K: Main Effects of Victimization Trajectories from SGD Status to Late Adolescent Health and Well-Being**

| Table S19 | | | | | | | |
| --- | --- | --- | --- | --- | --- | --- | --- |
| *Main Effects of SGD Status on Posterior Probabilities in Health and Well-Being Outcome Models* | | | | | | | |
| Health & well-being | SGD status | SGD → Posterior probabilities | | | | | |
|  |  | Early peak | | Adolescence onset | | Late childhood peak | |
|  |  | *β* | 95% CI | *β* | 95% CI | *β* | 95% CI |
| Self-harm | SA_D | .04*** | .02, .06 | .09*** | .07, .11 | .04*** | .02, .06 |
|  | SA_M | 0 | -.02, .02 | .03** | .01, .05 | .03** | .01, .05 |
|  | SI_D | .02 | 0, .04 | .10*** | .08, .12 | .05*** | .03, .07 |
|  | SI_M | 0 | -.02, .02 | .02 | 0, .04 | .02 | -.01, .04 |
|  | GD | 0 | -.02, .02 | .06*** | .04, .08 | 0 | -.02, .02 |
| Emotional problems | SA_D | .04*** | .02, .06 | .09*** | .07, .11 | .04*** | .02, .06 |
|  | SA_M | 0 | -.02, .02 | .03** | .01, .05 | .03** | .01, .05 |
|  | SI_D | .02 | 0, .04 | .10*** | .08, .12 | .05*** | .03, .07 |
|  | SI_M | 0 | -.02, .02 | .02* | 0, .04 | .02 | -.01, .04 |
|  | GD | 0 | -.02, .02 | .06*** | .04, .08 | 0 | -.02, .02 |
| Health | SA_D | .04*** | .02, .06 | .09*** | .07, .11 | .03*** | .02, .05 |
|  | SA_M | 0 | -.03, .02 | .02 | 0, .04 | .02* | 0, .04 |
|  | SI_D | .02* | 0, .04 | .10*** | .08, .12 | .04*** | .03, .06 |
|  | SI_M | 0 | -.03, .02 | .01 | -.01, .03 | .01 | -.01, .03 |
|  | GD | 0 | -.02, .02 | .06*** | .05, .08 | -.01 | -.03, .02 |
| Substance use | SA_D | .04*** | .02, .06 | .09*** | .07, .11 | .04*** | .02, .06 |
|  | SA_M | 0 | -.02, .02 | .03** | .01, .05 | .03** | .01, .05 |
|  | SI_D | .02 | 0, .04 | .10*** | .08, .12 | .05*** | .03, .07 |
|  | SI_M | 0 | -.02, .02 | .02* | 0, .04 | .02 | -.01, .04 |
|  | GD | 0 | -.02, .02 | .06*** | .04, .08 | 0 | -.02, .02 |
| *Note*. *SGD* Sexually and Gender Diverse, *SA_D* Sexual Attraction Diverse, *SA_M* Mostly Other-Sex Attracted, *SI_D* Sexual Identity Diverse, *SI_M* Mainly Heterosexual, *GD* Gender Diverse.  **p* < .05, ***p* < .01, ****p* < .001. | | | | | | | |

| Table S20 | | | | | | | | |
| --- | --- | --- | --- | --- | --- | --- | --- | --- |
| *Main Effects of Posterior Probabilities on Health and Well-Being Outcomes* | | | | | | | | |
| Health & well-being factors | | SO/  GI | Posterior probabilities → Outcomes | | | | | |
|  |  |  | Early peak | | Adolescence onset | | Late childhood peak | |
|  |  |  | *β* | 95% CI | *β* | 95% CI | *β* | 95% CI |
| Self-harm | | SA | .03** | .01, .05 | .12*** | .10, .14 | .03** | .01, .05 |
|  |  | SI | .03** | .01, .05 | .11*** | .09, .13 | .03** | .01, .05 |
|  |  | GI | .04*** | .02, .06 | .13*** | .11, .15 | .05*** | .03, .07 |
| Emotional problems | | SA | .03** | .01, .05 | .12*** | .10, .14 | .04*** | .03, .06 |
|  |  | SI | .03** | .01, .05 | .12*** | .10, .14 | .04*** | .02, .06 |
|  |  | GI | .04*** | .02, .06 | .14*** | .12, .16 | .06*** | .04, .08 |
| Health | General health | SA | -.02** | -.04, -.01 | -.12*** | -.13, -.10 | -.05*** | -.06, -.03 |
|  |  | SI | -.03* | -.04, -.01 | -.11*** | -.13, -.09 | -.05*** | -.06, -.03 |
|  |  | GI | -.03** | -.05, -.01 | -.12*** | -.14, -.10 | -.05*** | -.07, -.03 |
|  | Social & behavioral health concerns | SA | .01 | -.04, .06 | .27*** | .24, .30 | .06** | .02, .10 |
|  |  | SI | .02 | -.03, .06 | .27*** | .24, .30 | .05** | .02, .09 |
|  |  | GI | .02 | -.03, .07 | .28*** | .25, .32 | .06** | .02, .11 |
| Substance use | Smoking | SA | .02 | 0, .04 | .02* | 0, .04 | .03** | .01, .05 |
|  |  | SI | .02 | 0, .04 | .02 | 0, .04 | .03** | .01, .05 |
|  |  | GI | .02 | 0, .04 | .02* | 0, .04 | .04** | .02, .05 |
|  | Vaping | SA | -.02 | -.04, 0 | .03** | .01, .05 | .01 | -.01, .03 |
|  |  | SI | -.02 | -.04, 0 | .03** | .01, .05 | .01 | -.01, .03 |
|  |  | GI | -.02 | -.04, 0 | .04 | .02, .06 | .01 | -.01, .03 |
|  | Alcohol | SA | 0 | -.02, .02 | -.04*** | -.06, -.02 | -.03** | -.05, -.01 |
|  |  | SI | 0 | -.02, .02 | -.04*** | -.06, -.02 | -.03** | -.05, -.01 |
|  |  | GI | 0 | -.02, .02 | -.04*** | -.06, -.02 | -.03** | -.05, -.01 |
|  | Cannabis | SA | -.01 | -.03, .01 | -.04*** | -.06, -.02 | -.02 | -.04, 0 |
|  |  | SI | -.01 | -.03, .01 | -.04*** | -.07, -.02 | -.02 | -.04, 0 |
|  |  | GI | -.01 | -.03, .01 | -.04*** | -.06, -.02 | -.01 | -.03, .01 |
| *Note*. *SO/GI* Sexual Orientation and Gender Identity*, SA* Sexual Attraction, *SI* Sexual Identity, *GI* Gender Identity.  **p* < .05, ***p* < .01, ****p* < .001. | | | | | | | | |

# **Appendix L: The Pre-Registered Auxiliary Method**

| Table S21 | | | | | | | | |
| --- | --- | --- | --- | --- | --- | --- | --- | --- |
| *The Predictive Roles of SGD Statuses on Victimization Trajectories Using Different Methods* | | | | | | | | |
| SO/GI | Reference class | SGD status | Methods | | | | | |
|  |  |  | R3STEP | | | Class membership | | |
|  |  |  | Est. | *OR* | 95% CI | Est. | *OR* | 95% CI |
| Sexual attraction | Low |  | Early peak | | | | | |
|  |  | SA_D | 0.62*** | 1.87 | 1.41, 2.48 | 0.63^***^ | 1.88 | 1.49, 2.38 |
|  |  | SA_M | 0.07 | 1.07 | 0.80, 1.43 | 0.09 | 1.09 | 0.85, 1.40 |
|  |  |  | Late childhood peak | | | | | |
|  |  | SA_D | 0.72*** | 2.06 | 1.43, 2.98 | 0.58^***^ | 1.78 | 1.37, 2.32 |
|  |  | SA_M | 0.20 | 1.22 | 0.84, 1.77 | 0.23 | 1.25 | 0.97, 1.62 |
|  |  |  | Adolescence onset | | | | | |
|  |  | SA_D | 0.95*** | 2.57 | 2.08, 3.18 | 0.84^***^ | 2.32 | 1.94, 2.77 |
|  |  | SA_M | 0.36** | 1.43 | 1.16, 1.76 | 0.30^**^ | 1.35 | 1.14, 1.61 |
|  | Adolescence onset |  | Early peak | | | | | |
|  |  | SA_D | -0.32* | 0.73 | 0.53, 1.00 | -0.21 | 0.81 | 0.62, 1.06 |
|  |  | SA_M | -0.29 | 0.75 | 0.54, 1.05 | -0.22 | 0.81 | 0.61, 1.08 |
|  |  |  | Late childhood peak | | | | | |
|  |  | SA_D | -0.22 | 0.80 | 0.54, 1.19 | -0.27 | 0.77 | 0.57, 1.03 |
|  |  | SA_M | -0.16 | 0.85 | 0.57, 1.28 | -0.08 | 0.93 | 0.69, 1.24 |
|  | Late childhood peak |  | Early peak | | | | | |
|  |  | SA_D | -0.10 | 0.91 | 0.59, 1.39 | 0.05 | 1.06 | 0.76, 1.47 |
|  |  | SA_M | -0.13 | 0.88 | 0.56, 1.38 | -0.14 | 0.87 | 0.62, 1.23 |
| Sexual identity | Low |  | Early peak | | | | | |
|  |  | SI_D | 0.43** | 1.53 | 1.14, 2.07 | 0.45^***^ | 1.56 | 1.22, 20 |
|  |  | SI_M | -0.01 | 0.99 | 0.72, 1.35 | 0 | 1.00 | 0.76, 1.31 |
|  |  |  | Late childhood peak | | | | | |
|  |  | SI_D | 0.84*** | 2.31 | 1.63, 3.27 | 0.66^***^ | 1.94 | 1.50, 2.49 |
|  |  | SI_M | 0.11 | 1.12 | 0.73, 1.70 | 0.11 | 1.11 | 0.83, 1.48 |
|  |  |  | Adolescence onset | | | | | |
|  |  | SI_D | 0.99*** | 2.69 | 2.20, 3.30 | 0.87^***^ | 2.39 | 2.02, 2.84 |
|  |  | SI_M | 0.31** | 1.37 | 1.09, 1.72 | 0.23^*^ | 1.26 | 1.04, 1.53 |
|  | Adolescence onset |  | Early peak | | | | | |
|  |  | SI_D | -0.56** | 0.57 | 0.41, 0.79 | -0.43^**^ | 0.65 | 0.50, 0.86 |
|  |  | SI_M | -0.33 | 0.72 | 0.50, 1.04 | -0.24 | 0.79 | 0.58, 1.09 |
|  |  |  | Late childhood peak | | | | | |
|  |  | SI_D | -0.16 | 0.86 | 0.59, 1.24 | -0.21 | 0.81 | 0.61, 1.07 |
|  |  | SI_M | -0.20 | 0.82 | 0.52, 1.29 | -0.13 | 0.88 | 0.63, 1.22 |
|  | Late childhood peak |  | Early peak | | | | | |
|  |  | SI_D | -0.41 | 0.67 | 0.44, 1.02 | -0.22 | 0.81 | 0.58, 1.12 |
|  |  | SI_M | -0.12 | 0.89 | 0.53, 1.47 | -0.11 | 0.90 | 0.61, 1.32 |
| Gender identity | Low |  | Early peak | | | | | |
|  |  | GD | 0.54^+^ | 1.72 | 0.77, 3.87 | 0.47^+^ | 1.60 | 0.77, 3.32 |
|  |  |  | Late childhood peak | | | | | |
|  |  | GD | -0.50^+^ | 0.61 | 0.07, 5.03 | -0.08^+^ | 0.92 | 0.33, 2.58 |
|  |  |  | Adolescence onset | | | | | |
|  |  | GD | 1.35*** | 3.85 | 2.41, 6.13 | 1.23^***^ | 3.40 | 2.25, 5.16 |
| *Note. SO/GI* Sexual Orientation and Gender Identity, *SGD* Sexually and Gender Diverse, *SA_D* Sexual-attraction Diverse, *SA_M* Mostly Other-sex-attracted, *SI_D* Sexual-identity Diverse, *SI_M* Mainly Heterosexual, *GD* Gender Diverse. The predictive roles of SGD status are tested compared to heterosexual and cisgender youth. ^+^Due to the low numbers of gender diverse youth in the Early Peak and Late Childhood Peak classes, it is important to exercise caution when interpreting these results.  **p* < .05, ***p* < .01, ****p* < .001. | | | | | | | | |

# **Appendix M: Sensitivity Analyses Merging all SGD Subgroups**

Sensitivity analyses were conducted for all the main analyses, merging all the different SGD subgroups into one general SGD group. The results showed that being SGD, in general, was associated with heightened risks of being in the three victimized classes compared to the Low Victimization Class. Additionally, SGD identity was specifically linked to membership in the Adolescent Onset Class rather than the Early Peak Class. Such risks of prolonged peer bullying victimization were found to be related to SGD youth’s psychological and relational difficulties which can already emerge in early and middle childhood. Further, the higher probabilities of long-term bullying victimization during childhood and adolescence were linked to detrimental well-being in SGD youth during late adolescence, including increased self-harm behaviors, more emotional problems, and poorer health. It should be noted that merging all SGD subgroups into one general group helped achieve the largest subsample sizes, increasing the power to identify significant disparities. However, this approach may also mask the potential heterogeneity within the SGD population, who might experience varying levels of minority stress.

| Table S22 | | | | |
| --- | --- | --- | --- | --- |
| *The Predictive Role of Merged SGD Status on Victimization Trajectories* | | | | |
| Reference class | Membership Class | Est. | *OR* | 95% CI |
| Low | Early peak | 0.13** | 1.33 | 1.12, 1.59 |
|  | Late childhood peak | 0.16*** | 1.43 | 1.18, 1.73 |
|  | Adolescence onset | 0.24*** | 1.71 | 1.50, 1.95 |
| Adolescence onset | Early peak | -0.11* | 0.78 | 0.63, 0.96 |
|  | Late childhood peak | -0.08 | 0.84 | 0.67, 1.04 |
| Late childhood peak | Early peak | -0.03 | 0.93 | 0.73, 1.19 |
| *Note.* *SGD* Sexually and Gender Diverse.  **p* < .05, ***p* < .01, ****p* < .001. | | | | |

| Table S23 | | | | | | | | | | | | |
| --- | --- | --- | --- | --- | --- | --- | --- | --- | --- | --- | --- | --- |
| *Indirect Effects of Associated Mental Health and Relational Factors on the Relationship between the Merged SGD Status and Victimization Trajectories* | | | | | | | | | | | | |
| Mediator | Class membership | | Wave 2 | | Wave 3 | | Wave 4 | | Wave 5 | | Wave 6 | |
|  | Reference | Outcome | b | 95% CI | b | 95% CI | b | 95% CI | b | 95% CI | b | 95% CI |
| Emotional problems (Parent-report) | Low | EP | -0.03 | -0.06, 0.01 | 0.02 | -0.01, 0.04 | 0.04* | 0.02, 0.07 | 0.06* | 0.04, 0.08 | 0.09* | 0.06, 0.11 |
|  |  | LCP | -0.01 | -0.03, 0.00 | 0.01 | -0.01, 0.03 | 0.05* | 0.02, 0.08 | 0.09* | 0.05, 0.13 | 0.13* | 0.09, 0.17 |
|  |  | AO | -0.01 | -0.01, 0.00 | 0.01 | 0.00, 0.02 | 0.03* | 0.01, 0.05 | 0.08* | 0.05, 0.12 | 0.15* | 0.11, 0.19 |
|  | AO | EP | -0.02 | -0.05, 0.01 | 0.01 | -0.01, 0.02 | 0.01* | 0.00, 0.02 | -0.02* | -0.04, -0.01 | -0.06* | -0.09, -0.04 |
| Emotional problems (Youth-report) | Low | EP |  |  |  |  | 0 | -0.01, 0.01 | 0.04* | 0.02, 0.06 | 0.05* | 0.01, 0.08 |
|  |  | LCP |  |  |  |  | 0.02* | 0.01, 0.04 | 0.09* | 0.07, 0.12 | 0.11* | 0.07, 0.15 |
|  |  | AO |  |  |  |  | 0 | -0.01, 0.01 | 0.1* | 0.07, 0.12 | 0.16* | 0.13, 0.20 |
|  | AO | EP |  |  |  |  | 0 | -0.02, 0.01 | -0.06* | -0.09, -0.04 | -0.12* | -0.16, -0.07 |
| Hyperactive/inattention problems | Low | EP | 0 | -0.01, 0.02 | 0.03* | 0.01, 0.04 | 0.03* | 0.01, 0.04 | 0.03* | 0.02, 0.05 | 0.02* | 0.00, 0.03 |
|  |  | LCP | 0 | -0.01, 0.02 | 0.04* | 0.01, 0.06 | 0.05* | 0.02, 0.07 | 0.05* | 0.03, 0.08 | 0.02* | 0.00, 0.05 |
|  |  | AO | 0 | -0.01, 0.01 | 0.03* | 0.01, 0.05 | 0.04* | 0.02, 0.06 | 0.05* | 0.03, 0.08 | 0.03* | 0.00, 0.06 |
|  | AO | EP | 0 | 0.00, 0.00 | 0 | -0.01, 0.01 | -0.01* | -0.03, 0.00 | -0.02* | -0.03, -0.01 | -0.01 | -0.03, 0.00 |
| Self-esteem | Low | EP |  |  |  |  |  |  | 0.02* | 0.01, 0.03 | 0.05* | 0.03, 0.08 |
|  |  | LCP |  |  |  |  |  |  | 0.02* | 0.01, 0.04 | 0.05* | 0.02, 0.08 |
|  |  | AO |  |  |  |  |  |  | 0.04* | 0.02, 0.05 | 0.07* | 0.05, 0.09 |
|  | AO | EP |  |  |  |  |  |  | -0.02* | -0.03, 0.00 | -0.02 | -0.05, 0.02 |
| Peer problems  (Parent-report) | Low | EP | 0.04* | 0.01, 0.07 | 0.07* | 0.04, 0.09 | 0.09* | 0.07, 0.12 | 0.13* | 0.10, 0.16 | 0.16* | 0.13, 0.20 |
|  |  | LCP | 0.02* | 0.00, 0.03 | 0.06* | 0.04, 0.08 | 0.12* | 0.09, 0.16 | 0.19* | 0.14, 0.23 | 0.23* | 0.18, 0.27 |
|  |  | AO | 0.01* | 0.00, 0.02 | 0.04* | 0.03, 0.06 | 0.09* | 0.06, 0.11 | 0.18* | 0.14, 0.22 | 0.27* | 0.23, 0.32 |
|  | AO | EP | 0.03* | 0.01, 0.05 | 0.03* | 0.01, 0.04 | 0 | -0.01, 0.02 | -0.05* | -0.08, -0.03 | -0.11* | -0.15, -0.07 |
| Availability of friendships  (Youth-reported) | Low | EP |  |  |  |  | 0.01 | 0.00, 0.03 | 0.02* | 0.01, 0.03 | 0.01 | -0.01, 0.03 |
|  |  | LCP |  |  |  |  | 0.03* | 0.02, 0.05 | 0.02* | 0.01, 0.04 | 0.01 | -0.01, 0.03 |
|  |  | AO |  |  |  |  | 0.02* | 0.01, 0.03 | 0.03* | 0.02, 0.05 | 0.07* | 0.05, 0.09 |
|  | AO | EP |  |  |  |  | 0 | -0.02, 0.01 | -0.01 | -0.03, 0.00 | -0.05* | -0.08, -0.03 |
| Parent-child closeness (Parent-reported) | Low | EP |  |  | 0.01 | -0.01, 0.01 | 0 | 0.00, 0.01 | 0 | 0.00, 0.01 | 0.01* | 0.00, 0.02 |
|  |  | LCP |  |  | 0 | 0.00, 0.00 | 0 | 0.00, 0.01 | 0 | 0.00, 0.00 | 0 | 0.00, 0.01 |
|  |  | AO |  |  | 0 | 0.00, 0.00 | 0 | 0.00, 0.00 | 0 | 0.00, 0.01 | 0.01 | 0.00, 0.01 |
|  | AO | EP |  |  | 0 | 0.00, 0.01 | 0 | 0.00, 0.01 | 0 | 0.00, 0.00 | 0.01 | 0.00, 0.02 |
| Parent-child closeness (Youth-reported) | Low | EP |  |  |  |  |  |  |  |  | 0.03* | 0.01, 0.05 |
|  |  | LCP |  |  |  |  |  |  |  |  | 0.03* | 0.01, 0.05 |
|  |  | AO |  |  |  |  |  |  |  |  | 0.04* | 0.03, 0.06 |
|  | AO | EP |  |  |  |  |  |  |  |  | -0.01 | -0.03, 0.01 |
| Peer exclusion  (Youth-reported) | Low | EP |  |  |  |  | 0.01* | 0.00, 0.02 |  |  |  |  |
|  |  | LCP |  |  |  |  | 0.02* | 0.01, 0.03 |  |  |  |  |
|  |  | AO |  |  |  |  | 0.01* | 0.00, 0.02 |  |  |  |  |
|  | AO | EP |  |  |  |  | 0 | -0.01, 0.01 |  |  |  |  |
| Social disclosure (Youth-reported) | Low | EP |  |  |  |  | 0 | 0.00, 0.00 | 0 | 0.00, 0.01 | 0.01 | 0.00, 0.02 |
|  |  | LCP |  |  |  |  | 0 | 0.00, 0.00 | 0 | 0.00, 0.01 | 0.01 | 0.00, 0.02 |
|  |  | AO |  |  |  |  | 0 | 0.00, 0.00 | 0 | 0.00, 0.01 | 0.01* | 0.00, 0.02 |
|  | AO | EP |  |  |  |  | 0 | 0.00, 0.00 | 0 | -0.01, 0.00 | 0 | -0.02, 0.01 |
| Social support  (Youth reported) | Low | EP |  |  |  |  |  |  |  |  | 0.02* | 0.01, 0.04 |
|  |  | LCP |  |  |  |  |  |  |  |  | 0.03* | 0.01, 0.05 |
|  |  | AO |  |  |  |  |  |  |  |  | 0.04* | 0.02, 0.06 |
|  | AO | EP |  |  |  |  |  |  |  |  | -0.02 | -0.04, 0.00 |
| *Note*. *SGD* Sexually and Gender Diverse, *EP* Early Peak Victimization Class, *LCP* Late Childhood Peak Victimization Class, *AO* Adolescence Onset Victimization Class.  *Significant results after correcting for *α* level for multiple testing. | | | | | | | | | | | | |

| Table S24 | | | | | | | | | |
| --- | --- | --- | --- | --- | --- | --- | --- | --- | --- |
| *Main Effects of Merged SGD Status and Associated Factors, Using Low-Victimization Class as the Reference Class* | | | | | | | | | |
| Mediator | Wave | SGD → Mediator | | Mediator → EP | | Mediator → LCP | | Mediator → AO | |
|  |  | *β* | 95% CI | *β* | 95% CI | *β* | 95% CI | *β* | 95% CI |
| Emotional problems (Parent-reported) | 2 | -0.02 | -0.04, 0.00 | 0.71* | 0.64, 0.79 | 0.33* | 0.24, 0.42 | 0.12* | 0.05, 0.19 |
|  | 3 | 0.01 | -0.01, 0.03 | 0.55* | 0.47, 0.62 | 0.44* | 0.36, 0.53 | 0.25* | 0.19, 0.32 |
|  | 4 | 0.03* | 0.01, 0.06 | 0.56* | 0.48, 0.64 | 0.63* | 0.55, 0.71 | 0.4* | 0.34, 0.47 |
|  | 5 | 0.06* | 0.04, 0.08 | 0.47* | 0.39, 0.56 | 0.72* | 0.64, 0.81 | 0.67* | 0.61, 0.73 |
|  | 6 | 0.09* | 0.06, 0.11 | 0.45* | 0.36, 0.54 | 0.67* | 0.59, 0.76 | 0.78* | 0.71, 0.84 |
| Emotional problems (Youth-reported) | 4 | 0.05* | 0.03, 0.08 | 0.01 | -0.07, 0.10 | 0.17* | 0.07, 0.27 | 0.03 | -0.04, 0.11 |
|  | 5 | 0.11* | 0.09, 0.13 | 0.15* | 0.07, 0.23 | 0.39* | 0.31, 0.47 | 0.41* | 0.35, 0.47 |
|  | 6 | 0.2* | 0.17, 0.22 | 0.11* | 0.03, 0.19 | 0.24* | 0.15, 0.33 | 0.37* | 0.31, 0.43 |
| Hyperactive/inattention problems (Parent-reported) | 2 | 0 | -0.02, 0.02 | 0.31* | 0.23, 0.40 | 0.33* | 0.23, 0.42 | 0.27* | 0.20, 0.33 |
|  | 3 | 0.04* | 0.02, 0.06 | 0.34* | 0.27, 0.42 | 0.44* | 0.35, 0.53 | 0.35* | 0.29, 0.42 |
|  | 4 | 0.04* | 0.02, 0.06 | 0.3* | 0.21, 0.38 | 0.49* | 0.40, 0.58 | 0.44* | 0.38, 0.51 |
|  | 5 | 0.04* | 0.02, 0.06 | 0.33* | 0.24, 0.41 | 0.55* | 0.46, 0.63 | 0.54* | 0.47, 0.60 |
|  | 6 | 0.02* | 0.00, 0.04 | 0.31* | 0.23, 0.40 | 0.44* | 0.36, 0.53 | 0.55* | 0.48, 0.61 |
| Self-esteem (Youth-reported) | 5 | -0.06* | -0.08, -0.03 | -0.14* | -0.22, -0.05 | -0.2* | -0.28, -0.11 | -0.28* | -0.34, -0.22 |
|  | 6 | -0.14* | -0.16, -0.11 | -0.18* | -0.26, -0.09 | -0.17* | -0.26, -0.07 | -0.23* | -0.30, -0.16 |
| Peer problems (Parent-reported) | 2 | 0.03* | 0.01, 0.05 | 0.63* | 0.55, 0.71 | 0.29* | 0.20, 0.39 | 0.18* | 0.11, 0.25 |
|  | 3 | 0.07* | 0.04, 0.09 | 0.46* | 0.38, 0.54 | 0.42* | 0.33, 0.51 | 0.29* | 0.22, 0.35 |
|  | 4 | 0.08* | 0.06, 0.10 | 0.49* | 0.41, 0.57 | 0.66* | 0.58, 0.74 | 0.47* | 0.40, 0.54 |
|  | 5 | 0.11* | 0.09, 0.13 | 0.5* | 0.42, 0.58 | 0.74* | 0.65, 0.82 | 0.71* | 0.65, 0.78 |
|  | 6 | 0.15* | 0.13, 0.17 | 0.49* | 0.41, 0.58 | 0.69* | 0.60, 0.78 | 0.83* | 0.76, 0.90 |
| Availability of friendships (Youth-reported) | 4 | -0.07* | -0.09, -0.05 | -0.09 | -0.17, -0.01 | -0.2* | -0.29, -0.11 | -0.1* | -0.17, -0.04 |
|  | 5 | -0.07* | -0.10, -0.05 | -0.12* | -0.20, -0.04 | -0.14* | -0.22, -0.05 | -0.19* | -0.25, -0.13 |
|  | 6 | -0.11* | -0.13, -0.09 | -0.05 | -0.13, 0.03 | -0.04 | -0.14, 0.05 | -0.27* | -0.33, -0.20 |
| Peer exclusion (Youth-reported) | 4 | 0.04* | 0.02, 0.06 | 0.13* | 0.05, 0.22 | 0.22* | 0.12, 0.31 | 0.11* | 0.04, 0.18 |
| Parent-child closeness (Parent-reported) | 3 | -0.01 | -0.03, 0.01 | -0.19* | -0.26, -0.12 | 0.02 | -0.07, 0.12 | -0.06 | -0.12, 0.01 |
|  | 4 | -0.01 | -0.03, 0.01 | -0.16* | -0.23, -0.08 | -0.1 | -0.19, -0.01 | -0.05 | -0.12, 0.01 |
|  | 5 | -0.01 | -0.03, 0.01 | -0.06 | -0.14, 0.02 | -0.05 | -0.14, 0.05 | -0.07* | -0.14, -0.01 |
|  | 6 | -0.04* | -0.06, -0.02 | -0.16* | -0.24, -0.08 | -0.05 | -0.14, 0.04 | -0.08* | -0.14, -0.01 |
| Parent-child closeness (Youth-reported) | 6 | -0.09* | -0.12, -0.07 | -0.15* | -0.24, -0.07 | -0.13* | -0.22, -0.04 | -0.2* | -0.26, -0.13 |
| Social disclosure (Youth-reported) | 4 | -0.01 | -0.03, 0.01 | 0 | -0.08, 0.09 | -0.01 | -0.10, 0.08 | -0.01 | -0.08, 0.05 |
|  | 5 | -0.03* | -0.05, -0.01 | -0.02 | -0.10, 0.07 | -0.05 | -0.13, 0.03 | -0.06 | -0.12, 0.00 |
|  | 6 | -0.07* | -0.09, -0.05 | -0.07 | -0.15, 0.02 | -0.07 | -0.15, 0.02 | -0.08* | -0.15, -0.02 |
| Social support (Youth reported) | 6 | -0.1* | -0.13, -0.08 | -0.1* | -0.17, -0.02 | -0.15* | -0.23, -0.07 | -0.17* | -0.23, -0.11 |
| *Note*. *SGD* Sexually and Gender Diverse, *EP* Early Peak Victimization Class, *LCP* Late Childhood Peak Victimization Class, *AO* Adolescence Onset Victimization Class.  *Significant results after correcting for α level for multiple testing. | | | | | | | | | |

| Table S25 | | | | | |
| --- | --- | --- | --- | --- | --- |
| *Main Effects of Merged SGD Status and Associated Factors, Using Adolescence Onset Victimization Class as the Reference Class* | | | | | |
| Mediator | Wave | SGD → Mediator | | Mediator → EP | |
|  |  | *β* | 95% CI | *β* | 95% CI |
| Emotional problems (Parent-reported) | 2 | -0.02 | -0.04, 0.00 | -0.12* | -0.19, -0.05 |
|  | 3 | 0.01 | -0.01, 0.03 | -0.25* | -0.32, -0.19 |
|  | 4 | 0.03* | 0.01, 0.06 | -0.4* | -0.47, -0.34 |
|  | 5 | 0.06* | 0.04, 0.08 | -0.67* | -0.73, -0.61 |
|  | 6 | 0.09* | 0.06, 0.11 | -0.78* | -0.84, -0.71 |
| Emotional problems (Youth-reported) | 4 | 0.05* | 0.03, 0.08 | -0.03 | -0.11, 0.04 |
|  | 5 | 0.11* | 0.09, 0.13 | -0.41* | -0.47, -0.35 |
|  | 6 | 0.2* | 0.17, 0.22 | -0.37* | -0.43, -0.31 |
| Hyperactive/inattention problems  (Parent-reported) | 2 | 0 | -0.02, 0.02 | -0.27* | -0.33, -0.20 |
|  | 3 | 0.04* | 0.02, 0.06 | -0.35* | -0.42, -0.29 |
|  | 4 | 0.04* | 0.02, 0.06 | -0.44* | -0.51, -0.38 |
|  | 5 | 0.04* | 0.02, 0.06 | -0.54* | -0.60, -0.47 |
|  | 6 | 0.02* | 0.00, 0.04 | -0.55* | -0.61, -0.48 |
| Self-esteem  (Youth-reported) | 5 | -0.06* | -0.08, -0.03 | 0.28* | 0.22, 0.34 |
|  | 6 | -0.14* | -0.16, -0.11 | 0.23* | 0.16, 0.30 |
| Peer problems  (Parent-reported) | 2 | 0.03* | 0.01, 0.05 | -0.18* | -0.25, -0.11 |
|  | 3 | 0.07* | 0.04, 0.09 | -0.29* | -0.35, -0.22 |
|  | 4 | 0.08* | 0.06, 0.10 | -0.47* | -0.54, -0.40 |
|  | 5 | 0.11* | 0.09, 0.13 | -0.71* | -0.78, -0.65 |
|  | 6 | 0.15* | 0.13, 0.17 | -0.83* | -0.90, -0.76 |
| Availability of friendships  (Youth-reported) | 4 | -0.07* | -0.09, -0.05 | 0.1* | 0.04, 0.17 |
|  | 5 | -0.07* | -0.10, -0.05 | 0.19* | 0.13, 0.25 |
|  | 6 | -0.11* | -0.13, -0.09 | 0.27* | 0.20, 0.33 |
| Peer exclusion  (Youth-reported) | 4 | 0.04* | 0.02, 0.06 | -0.11* | -0.18, -0.04 |
| Parent-child closeness (Parent-reported) | 3 | -0.01 | -0.03, 0.01 | 0.06 | -0.01, 0.12 |
|  | 4 | -0.01 | -0.03, 0.01 | 0.05 | -0.01, 0.12 |
|  | 5 | -0.01 | -0.03, 0.01 | 0.07* | 0.01, 0.14 |
|  | 6 | -0.04* | -0.06, -0.02 | 0.08* | 0.01, 0.14 |
| Parent-child closeness (Youth-reported) | 6 | -0.09* | -0.12, -0.07 | 0.2* | 0.13, 0.26 |
| Social disclosure (Youth-reported) | 4 | -0.01 | -0.03, 0.01 | 0.01 | -0.05, 0.08 |
|  | 5 | -0.03* | -0.05, -0.01 | 0.06 | 0.00, 0.12 |
|  | 6 | -0.07* | -0.09, -0.05 | 0.08* | 0.02, 0.15 |
| Social support  (Youth reported) | 6 | -0.1* | -0.13, -0.08 | 0.17* | 0.11, 0.23 |
| *Note.* *SGD* Sexually and Gender Diverse, *M* Mediator, *EP* Early Peak Victimization Class, *LCP* Late Childhood Peak Victimization Class, *AO* Adolescence Onset Victimization Class.  *Significant results after correcting for α level for multiple testing. | | | | | |

| Table S26 | | | | | | | | | |
| --- | --- | --- | --- | --- | --- | --- | --- | --- | --- |
| *Effects of Merged SGD Status on Late Adolescent Health and Well-Being through Victimization Trajectories* | | | | | | | | | |
| Health & well-being factors | | SGD → Health/Well-being | | Indirect effect (via posterior probabilities) | | | | | |
|  |  |  |  | Early peak | | Adolescence onset | | Late childhood peak | |
|  |  | *β* | 95% CI | b | 95% CI | b | 95% CI | b | 95% CI |
| Self-harm | | 0.26*** | 0.24, 0.28 | 0 | 0.00, 0.00 | 0.02*** | 0.01, 0.03 | 0* | 0.00, 0.01 |
| Emotional problems | | 0.27*** | 0.25, 0.28 | 0 | 0.00, 0.00 | 0.02*** | 0.01, 0.02 | 0** | 0.00, 0.01 |
| Health | General health | -0.13*** | -0.14, -0.11 | 0 | 0.00, 0.00 | -0.02*** | -0.02, -0.01 | 0* | 0.00, 0.00 |
|  | Social & behavioral health concerns | 0.17*** | 0.12, 0.21 | 0 | 0.00, 0.00 | 0.05*** | 0.04, 0.06 | 0 | 0.00, 0.01 |
| Substance use | Smoking | 0.02 | 0.00, 0.04 | 0 | 0.00, 0.00 | 0 | 0.00, 0.00 | 0* | 0.00, 0.00 |
|  | Vaping | 0.02* | 0.00, 0.04 | 0 | 0.00, 0.00 | 0** | 0.00, 0.00 | 0 | 0.00, 0.00 |
|  | Alcohol | 0.01 | -0.01, 0.03 | 0 | 0.00, 0.00 | 0** | 0.00, 0.00 | 0* | 0.00, 0.00 |
|  | Cannabis | 0.09*** | 0.07, 0.11 | 0 | 0.00, 0.00 | 0*** | -0.01, 0.00 | 0 | 0.00, 0.00 |
| *Note.* *SGD* Sexually and Gender Diverse. In this table, main effect estimates (*β*) are presented as standardized coefficients, while the indirect effect estimates (b) are reported as unstandardized coefficients.  **p* < .05, ***p* < .01, ****p* < .001. | | | | | | | | | |

| Table S27 | | | | | | | |
| --- | --- | --- | --- | --- | --- | --- | --- |
| *Effects of Merged SGD Status on Victimization Trajectories, and Victimization Trajectories on Health and Well-Being* | | | | | | | |
| Health & well-being factors | | SGD → Posterior probabilities | | | | | |
|  |  | Early peak | | Adolescence onset | | Late childhood peak | |
|  |  | *β* | 95% CI | *β* | 95% CI | *β* | 95% CI |
| Self-harm | | 0.02* | 0.00, 0.04 | 0.08*** | 0.06, 0.10 | 0.04*** | 0.02, 0.06 |
| Emotional problems | | 0.02* | 0.00, 0.04 | 0.08*** | 0.06, 0.10 | 0.04*** | 0.02, 0.06 |
| Health | | 0.01 | -0.01, 0.03 | 0.08*** | 0.06, 0.10 | 0.03** | 0.01, 0.05 |
| Substance use | | 0.02* | 0.00, 0.04 | 0.08*** | 0.06, 0.10 | 0.04*** | 0.02, 0.06 |
| Health & well-being factors | | Posterior probabilities → Outcome | | | | | |
|  |  | Early peak | | Adolescence onset | | Late childhood peak | |
|  |  | *β* | 95% CI | *β* | 95% CI | *β* | 95% CI |
| Self-harm | | 0.03** | 0.01, 0.05 | 0.12*** | 0.10, 0.14 | 0.03** | 0.01, 0.05 |
| Emotional problems | | 0.03** | 0.01, 0.05 | 0.12*** | 0.11, 0.14 | 0.04*** | 0.03, 0.06 |
| Health | General health | -0.03* | -0.04, -0.01 | -0.11*** | -0.13, -0.10 | -0.05*** | -0.06, -0.03 |
|  | Social & behavioral health concerns | 0.01 | -0.03, 0.06 | 0.27*** | 0.24, 0.31 | 0.05** | 0.02, 0.09 |
| Substance use | Smoking | 0.02 | 0.00, 0.04 | 0.02* | 0.00, 0.04 | 0.03** | 0.01, 0.05 |
|  | Vaping | -0.02 | -0.04, 0.00 | 0.03** | 0.01, 0.05 | 0.01 | -0.01, 0.03 |
|  | Alcohol | 0 | -0.02, 0.02 | -0.04*** | -0.06, -0.02 | -0.03** | -0.05, -0.01 |
|  | Cannabis | -0.02 | -0.04, 0.01 | -0.05*** | -0.07, -0.02 | -0.02 | -0.04, 0.00 |
| Note. *SGD* Sexually and Gender Diverse.  **p* < .05, ***p* < .01, ****p* < .001. | | | | | | | |

# **Appendix N: Cross-Informant Comparisons Between Parent- and Youth-reported Victimization**

**1 Measurement**

In the Millennium Cohort Study (MCS) data, youth’s self-reported victimization items were available starting from Wave 4. However, the detailed measurement changed across waves, and none of the self-reported measures were consistent with parent-reported peer bullying victimization used in the current study. Table 1 displays the cross-informant victimization measures in the MCS data.

| Table S28 | | | | | | | | |
| --- | --- | --- | --- | --- | --- | --- | --- | --- |
| *Victimization Measurement in the MCS Data* | | | | | | | | |
| Variable | Wave 2 | Wave 3 | Wave 4 | Wave 5 | Wave 6 | Wave 7 | Item | Scale |
| Peer bullying victimization  (Parent-reported) | Agreement | Agreement | Agreement | Agreement | Agreement | Agreement | (Kid's name) is picked on or bullied by other children. | 3-point; 1 = *Not true* to 3 = *Very true* |
| Peer bullying victimization  (Youth-reported) |  |  | Frequency |  |  |  | How often do other children bully you? | 3-point; 0 = *Never* to 1 = *All of the time* |
| (General) Peer victimization  (Youth-reported) |  |  |  | Frequency | Frequency |  | How often do other children hurt you or pick on you on purpose? | 6-point; 0 = *Never* to 5 =*Most days* |
| General victimization (Youth-reported) |  |  |  |  | Yes/No | Yes/No | 5 items at Wave 6 (e.g., *Been physically violent towards you*; *Hit you with or used a weapon against you*);  9 items at Wave 7 (e.g., *Spread gossip about you*; *Assaulted you sexually*) | 2-point; 0 = *No* and 1 = *Yes* |

**2 Crosstabulation**

Given the inconsistent measurement and the skewed distribution of responses, the cross-informant victimization measures were coded into binary variables as 0 indicating *not victimized* and 1 for *victimized*, and the crosstabulations were tested. A crosstabulation analysis was conducted between cross-informant victimization status and SGD status (see Table 2).

Further, the consistency between informants was calculated based on the crosstabulation: cases where youth reported being victimized but parents did not were calculated as “*under-report*”, and conversely, cases where youth reported no victimization, but parents reported victimization were calculated as “*false report*” (see Table 3). As shown, across the overall sample, over one-third of parents tended to under-report their children's experiences of victimization, with a notably high rate of 46% at Wave 7 (age 17). Meanwhile, around 5% to 7% of parents falsely detected their children’s victimization. Seeing the intragroup proportions, that is, within both the SGD and non-SGD populations, except at Wave 4 (age 7), cross-informant consistency was generally lower among SGD youth compared to heterosexual, cisgender youth. It is important to note that the observed inconsistencies between parent- and youth-reported victimization, as calculated in this section, reflected not only the parents' ability to accurately identify their children’s victimization experiences but also the potential biases caused by the different measures.

| Table S29 | | | | | | | | |
| --- | --- | --- | --- | --- | --- | --- | --- | --- |
| *Crosstabulations Between Youth- and Parent-Reported Victimization* | | | | | | | | |
| SGD status | Youth-report | | Peer bullying (Parent-report) | | | | | |
|  |  |  | *n* | | Overall % | | Intragroup % | |
|  |  |  | No | Vic | No | Vic | No | Vic |
| Non-SGD | Wave 4  Peer bullying  (*n* = 8260) | No | 2802 | 352 | 33.9% | 4.3% | 46.6% | 5.8% |
|  |  | Vic | 2087 | 777 | 25.3% | 9.4% | 34.7% | 12.9% |
| SGD |  | No | 1017 | 137 | 12.3% | 1.7% | 45.4% | 6.1% |
|  |  | Vic | 754 | 334 | 9.1% | 4.0% | 33.6% | 14.9% |
| Non-SGD | Wave 5  Peer victimization  (*n* = 8846) | No | 2546 | 326 | 28.8% | 3.7% | 39.4% | 5.0% |
|  |  | Vic | 2473 | 1112 | 28.0% | 12.6% | 38.3% | 17.2% |
| SGD |  | No | 737 | 131 | 8.3% | 1.5% | 30.8% | 5.5% |
|  |  | Vic | 968 | 553 | 10.9% | 6.3% | 40.5% | 23.1% |
| Non-SGD | Wave 6  Peer victimization  (*n* = 8755) | No | 3006 | 455 | 34.3% | 5.2% | 47.2% | 7.2% |
|  |  | Vic | 2082 | 820 | 23.8% | 9.4% | 32.7% | 12.9% |
| SGD |  | No | 755 | 180 | 8.6% | 2.1% | 31.6% | 7.5% |
|  |  | Vic | 969 | 488 | 11.1% | 5.6% | 40.5% | 20.4% |
| Non-SGD | Wave 6  General victimization  (*n* = 8762) | No | 3010 | 448 | 34.4% | 5.1% | 47.3% | 7.0% |
|  |  | Vic | 2085 | 827 | 23.8% | 9.4% | 32.7% | 13.0% |
| SGD |  | No | 806 | 212 | 9.2% | 2.4% | 33.7% | 8.9% |
|  |  | Vic | 918 | 456 | 10.5% | 5.2% | 38.4% | 19.1% |
| Non-SGD | Wave 7  General victimization  (*n* = 7853) | No | 2473 | 278 | 31.5% | 3.5% | 43.3% | 4.9% |
|  |  | Vic | 2477 | 489 | 31.5% | 6.2% | 43.3% | 8.6% |
| SGD |  | No | 550 | 95 | 7.0% | 1.2% | 25.7% | 4.4% |
|  |  | Vic | 1145 | 346 | 14.6% | 4.4% | 53.6% | 16.2% |
| *Note*. *SGD* Sexually and Gender Diverse; *No* Not Victimized, *Vic* Victimized. Listwise deletion was conducted for crosstabulation analysis. | | | | | | | | |
